# Cognitive and social well-being in older adulthood: The CoSoWELL corpus of written life stories

**DOI:** 10.3758/s13428-022-01926-0

**Published:** 2022-08-24

**Authors:** Aki-Juhani Kyröläinen, James Gillett, Megan Karabin, Ranil Sonnadara, Victor Kuperman

**Affiliations:** 1 Department of Linguistics and Languages, McMaster University, Togo Salmon Hall 513, 1280 Main Street West, Hamilton, Ontario Canada 8S 4M2; 2 McMaster University, Hamilton, Canada

**Keywords:** Aging, Autobiographical memory, Text analytics, COVID-19

## Abstract

This paper presents the Cognitive and Social WELL-being (CoSoWELL) project that consists of two components. One is a large corpus of narratives written by over 1000 North American older adults (55+ years old) in five test sessions before and during the first year of the COVID-19 pandemic. The other component is a rich collection of socio-demographic data collected through a survey from the same participants. This paper introduces the first release of the corpus consisting of 1.3 million tokens and the survey data (CoSoWELL version 1.0). It also presents a series of analyses validating design decisions for creating the corpus of narratives written about personal life events that took place in the distant past, recent past (yesterday) and future, along with control narratives. We report results of computational topic modeling and linguistic analyses of the narratives in the corpus, which track the time-locked impact of the COVID-19 pandemic on the content of autobiographical memories before and during the COVID-19 pandemic. The main findings demonstrate a high validity of our analytical approach to unique narrative data and point to both the locus of topical shifts (narratives about recent past and future) and their detailed timeline. We make the CoSoWELL corpus and survey data available to researchers and discuss implications of our findings in the framework of research on aging and autobiographical memories under stress.

**Supplementary Information:**

The online version contains supplementary material available at 10.3758/s13428-022-01926-0.

## Introduction

At the time of this writing, most of humankind finds itself part of a global uncontrolled psychological experiment. Due to the COVID-19 pandemic and protective counter-measures, daily lives of most individuals and societies in the world have been experiencing a profound and prolonged disruption. An important goal for psychological science is to document the changes in human psychology and behavior brought about by this disruption. This paper focuses on language behavior before and during COVID-19 of one of the most vulnerable segments of world population, i.e., older adults. The impact of COVID-19 on older adults is particularly detrimental, with an increased risk of death compared to younger age groups (Ho et al., 2020; Shahid et al., 2020). Even if their physical health remained unaffected, older individuals as a group have been argued to carry a heavy psychological burden of physical and social isolation in the course of the pandemic (Brooks et al., 2020; Holmes et al., 2020). A promising avenue to charting the unfolding impact of the pandemic on the mental well-being of older adults is through documenting and analyzing their language use. Language production is known to reflect the mental and emotional state of the speaker/writer (e.g., Boyd et al., 2015; Mairesse, Walker, Mehl, & Moore, 2007; Schwartz et al., 2013; Yarkoni, 2010), as well as the relative importance of the themes that are at the forefront of their attention (e.g., Cohn, Mehl, & Pennebaker, 2004; Pennebaker & King, 1999; Stirman & Pennebaker, 2001). Patterns of language use have also been shown to serve as indicators of depression (Eichstaedt et al., 2018), dementia, and Alzheimer’s disease (Eyigoz, Mathur, Santamaria, Cecchi, & Naylor, 2020) among other issues of relevance for psychological well-being. Our project creates a resource that enables social scientists to examine patterns of language change as indices of psychological processes associated with the onset and progression of the COVID-19 pandemic.

This paper presents the Cognitive and Social WELL-being (CoSoWELL) corpus, i.e., a large collection of narratives written by older adults (55+ years old) before and during the first year of the pandemic, further supplemented by rich demographic and psychological participant survey data. The focus of this paper is on introducing the design and data of the CoSoWELL corpus, the pipeline used for the quantitative analysis of stories, and the validity of this resource for the multi-faceted time-locked study of the psychological outcomes of the pandemic in older adulthood. We also highlight the expected utility of the CoSoWELL corpus for memory-related studies relying on narrative language use (e.g., Conway, 2005; Fivush, 2011) as well the field of narrative gerontology (Bjursell, 2019; Randall, Kenyon, & Gary, 2004).

The CoSoWELL project consists of two components: a collection of elicited personal, written narratives and an extensive demographic and psychological survey obtained from the same participants. The conception and design of the CoSoWELL project as well as initial data collection predate the COVID-19 pandemic. Thus, the goals and the anticipated use of these data go beyond characterizing psychological outcomes of the pandemic. Specifically, the comprehensive goal for the project, which the present paper introduces, is to study the change in language use as a function of age, social isolation and perceived loneliness of older adults, using their personal life stories as a window into their memory functioning, and mental and emotional well-being. Importantly, language as a system is forged in social interactions that are, at least partly, grounded in storytelling and our ability to recall and communicate memories from our personal life (Chafe, 1994; Labov, 1972). Thus, language both mirrors and shapes the world of an individual (Yarkoni, 2010; Trope & Liberman, 2003; Semin & Smith, 1999).

Personal life stories stand out from other forms of language production in that they provide a rich source for both documenting language use and understanding its link to the psychological state of the story-teller (e.g., Conway, 2005; Fivush, 2011). This type of data is inherently connected to the cognitive basis of memories. Importantly, researchers agree that autobiographical memory does not merely store and reflect facts of one’s lived experience but rather it constitutes a subjective perspective that defines an individual self in interaction with other individuals and provides a sense of self-continuity (Conway, 2005; Fivush, 2011; Prebble, Addis, & Tippett, 2013). Not only is autobiographical memory engaged in the act of sharing one’s perspective via storytelling, but this memory is also critical for developing intimacy and maintaining social relationships (Alea & Bluck, 2003). While building on specific episodic and semantic memories of the “where”, “when” and “what” of occurring events (Tulving, 1972; 1983; for an overview see Renoult & Rugg, 2020), autobiographic memory links these events together, forming a personal narrative of lived experiences.

Specifically, autobiographical events in the distant past tend to portray constitutive moments in one’s life among younger (e.g., McAdams et al., 2006) and older adults (e.g., Thomsen, Pillemer, & Ivcevic, 2011). Additionally, they tend to include events that have been shared numerous times in social interactions. From this perspective, the content of these narratives (labeled as story type “past”) can be understood as representing rehearsed language. Personal narratives about the past tend to bring about similar, recurrent themes such as family relations and education that we use to anchor our past experiences to form a sense of self (Shum, 1998; Rathbone, Holmes, Murphy, & Ellis, 2015; Thomsen & Berntsen, 2008). Conversely, narratives about the recent past (story type “yesterday”) are typically unrehearsed and, during stable periods, reflect everyday repetitive activities and events including acts of communication and socialization, preparing meals, and shopping, among other things. These events tend to be culturally shared and easily identifiable. However, abrupt changes to this stability as presented by the global pandemic can lead to changes in the perception of individual’s life as the everyday life begins to include numerous unprecedented one-off events and even completely new experiences. If the period of instability is prolonged before new normalcy is established, an individual’s perception of one’s life experience may undergo a gradual shift. Furthermore, the autobiographical memory system enables projecting oneself into the future, i.e., a ‘mental time traveling’ (Bartlett, 1932; Tulving, 1985). Already at an early age, children are capable of imagining future life events and these projected, hypothetical events tend to be culturally shared (e.g., Atance & O’Neill, 2005).

In general, all types of autobiographical memories are strongly driven by socio-cultural factors and they continue to develop with advancing age (Bohn & Berntsen, 2011; Donald, 2001; Nelson & Fivush, 2004; Kenyon, Gar, & Randall, 1999). Indeed, language is the de facto vehicle at our disposal to organize and share experiences. From this perspective, narratives themselves constitute learned patterns that govern our perception of the events unfolding in time—from the distant past to the recent past to the future—and provide the glue for linking the individual events into a coherent sequence (e.g., Chafe, 1980; 1994; Labov, 1972; Labov & Waletzky, 1967; Linde & et al. 1993). The key part that autobiographical memories play in defining a person’s identity and the link of these stories to such linguistic genres as story-telling motivate the choice of personal life stories as the central content of the CoSoWELL project.

The CoSoWELL project is uniquely positioned to pursue both its original goal—investigating how language use can reflect differences in age, social isolation and loneliness—and the new direction dictated by the COVID-19 pandemic. The corpus data collection was initially conducted in March–June 2019 with the original goal in sights. However, soon after the onset of COVID-19 in North America, this project was revisited to factor in this new challenge to psychological well-being. The pre-pandemic 2019 data were re-designated as the first test session (t1) of a time-series and an additional four test sessions were administered (t2–t5).

We conducted data collection using the same stimuli and partly overlapping participant pool starting April 08, 2020 (3 weeks after the global lockdown in North America) and until June 16, 2020. We will refer to this test session as (t2). The third test session (t3) lasted from June 17 through June 30, 2020, roughly 4 months after the large-scale onset of the pandemic in the US and Canada and nation-wide counter-measures. At that time, a slight decrease in the number of new cases and deaths was observed in the US and Canada, leading to a temporary weakening of the lockdown that led into a spike in the number of COVID-19 cases later in the fall. The fourth test session (t4) started October 14 and ended on November 5, 2020. The number of deaths related to COVID-19 rose in the United States and crossed 500,000, the highest reported in the world, on February 22, 2021 (https://covid.cdc.gov/covid-data-tracker/#trends_totalandratedeathssevendayrate). Our fifth test session (t5) covered this time period as it commenced on January 12, 2021, and continued until February 15, 2021. Informally, we consider data from t1 as a pre-pandemic baseline, data from t2 as behavioral outcomes originating from the initial public exposure to COVID-19 and national response to it, and data from t3 to t5 as behavioral snapshots tracking the timeline of COVID-19. The number of COVID-19 cases and deaths in the United States and Canada during the period of the data collection are summarized in Table 1 based on the information provided at https://covid19.who.int/region/amro/country/us and https://covid19.who.int/region/amro/country/ca.

**Table 1:** Number of COVID-19 cases and deaths in the United States and Canada

USA		CANADA	
T1 (March 1, 2019 to June 30, 2019)			
Total confirmed cases as of March 1, 2019	0	Total confirmed cases as of March 1, 2019	0
Total confirmed deaths as of March 1, 2019	0	Total confirmed deaths as of March 1, 2019	0
T2 (April 8, 2020 to June 16, 2020)			
Total confirmed cases as of April 8, 2020	378,220	Total confirmed cases as of April 8, 2020	17,049
Total confirmed deaths as of April 8, 2020	12,620	Total confirmed deaths as of April 8, 2020	345
Week of March 30 – April 5 cases	171,279	Week of March 30 – April 5 cases	8181
Week of March 30 – April 5 deaths	5002	Week of March 30 – April 5 deaths	159
T3 (June 17, 2020 to June 30, 2020)			
Total confirmed cases as of June 17, 2020	2,105,922	Total confirmed cases as of June 17, 2020	108,829
Total confirmed deaths as of June 17, 2020	117,182	Total confirmed deaths as of June 17, 2020	8175
Week of July 13 – July 19 cases	461,531	Week of July 13 – July 19 cases	2543
Week of July 13 – July 19 deaths	6263	Week of July 13 – July 19 deaths	80
T4 (Oct 14, 2020 to Nov 5, 2020)			
Total confirmed cases as of Oct 14, 2020	7,880,896	Total confirmed cases as of October 14, 2020	182,839
Total confirmed deaths as of Oct 14, 2020	219,658	Total confirmed deaths as of October 14, 2020	9589
Week of Oct 12 – Oct 19 cases	387,466	Week of Oct 12 – Oct 19 cases	15,989
Week of Oct 12 – Oct 19 deaths	4805	Week of Oct 12 – Oct 19 deaths	136
T5 (Jan 12, 2021 to Feb 15, 2021)			
Total confirmed cases as of Jan 12, 2021	23,533,768	Total confirmed cases as of Jan 12, 2021	661,334
Total confirmed deaths as of Jan 12, 2021	390,029	Total confirmed deaths as of Jan 12, 2021	16,849
Week of Jan 11 – Jan 17 cases	1,591,103	Week of Jan 11 – Jan 17 cases	51,355
Week of Jan 11 – Jan 17 deaths	23,289	Week of Jan 11 – Jan 17 deaths	1016

The current release of the CoSoWELL project (version 1.0) presents a large collection of written narratives consisting of over 1.3 million tokens produced by 1178 unique participants covering four story types. Of these individuals, 1028 also completed the survey (see below). Three of the story types constitute stories about personal life events thematically anchored across the entire timeline: namely, distant past, yesterday, and future. These story types were specifically chosen to elicit life stories that provide the possibility of mental time travel through personal life experiences and engage different facets of autobiographical memories. The fourth and final story type served as a control and was elicited based on a picture from the Boston Diagnostic Aphasia Examination depicting the Cookie Theft scene (Goodglass, Kaplan, & Barresi, 2001). Language patterns elicited in the description of this standard picture in clinical settings (for an overview see Cummings, 2019) have been demonstrated to display sensitivity to language impairments in addition to aphasia such as frontotemporal dementia (Ash et al., 2016), Alzheimer’s disease (e.g., Eyigoz et al., 2020), and right-hemisphere stroke and left-hemisphere stroke (Agis et al., 2016).

In our study, this control condition ensured that all participants at all time points were tasked to provide a written narrative from the same topic. This provided a baseline of linguistic patterns both within and across individual participants and test sessions. In the remainder of the paper, we refer to elicited autobiographical stories about the distant past, yesterday, and future as *life stories* and we collectively refer to all stories in the CoSoWELL corpus, regardless of their story type, as narratives.

In addition to the corpus data, the CoSoWELL project also includes an extensive survey study that consists of questions pertaining to educational background, language use and habits as well as social and cognitive well-being. The goal of the survey study was two-fold: (1) to enrich and supplement analyses based on the written narratives reflecting autobiographical memories and (2) to provide a detailed and comprehensive profile of an individual pertaining to their social and cognitive well-being. The survey itself can be understood as consisting of two parts. The first one pertains to an individual’s background information. The questions used in this part of the CoSoWELL survey were partially based on previous research on aging, including the Cognitive Reserve scale (Nucci, Mapelli, & Mondini, 2012) and instruments used in large-scale studies such as the Survey on Ageing and Independence (https://www23.statcan.gc.ca/imdb/p2SV.pl?Function=getSurvey&SDDS=&3885) and the Canadian Longitudinal Study on Aging (Raina et al., 2019). The second part of the CoSoWELL survey consists of the instrument designed to measure social and cognitive well-being: i.e., the three-item loneliness scale (Hughes, Waite, Hawkley, & Cacioppo, 2004) and the Prospective-Retrospective Memory Questionnaire (Smith, Sala, Logie, & Maylor, 2000). These instruments will be released as part of the version 2 release of the CoSoWELL project. The survey was completed by 1451 participants, of whom 1028 have also completed the narrative writing task. In the current release of the CoSoWELL project, we publish partial survey data representing 39 variables from the 1451 participants in the survey. The survey data can either be linked to the CoSoWELL corpus data (for *n* = 1028 participants) to examine their written productions jointly with their socio-demographic data, or it can be explored independently, as a snapshot of social and psychological variables collected during the pandemic. In our view, this project provides a unique opportunity to investigate the intersection of language use and social and cognitive well-being in older adulthood and, importantly, during the COVID-19 pandemic.

CoSoWELL is an ongoing project, with at least three additional test periods of data collection planned to enable continuous tracking of language behavior throughout and after the pandemic. The present paper reports processed and annotated text data that CoSoWELL comprises so far (from t1 to t5), along with basic demographic information on the participants that can be linked to the stories. Future releases of the corpus will add newly collected data (from t6 and t8) and also update the current materials by adding annotations or correcting minor imperfections. We relegate to the future the publication of collected survey data on perceived loneliness, social isolation, and memory functioning. Given the rich nature of the linguistic, demographic, and other data in CoSoWELL, no single report can pursue all analytical possibilities that this resource offers. The present, first paper on the project specifically focuses on the methodology employed in creating the CoSoWELL corpus and its validity as a research resource. Below we point to additional directions of academic and applied interest that future research can follow up on.

The objectives of the current paper are two-fold. First, we provide social scientists with a unique corpus of English narratives written by North American older adults before and during the pandemic. The collection of the narratives provided in the corpus is enhanced by rich lexical and syntactic annotation at the word level, as well as the demographic and psychological participant meta-data from the CoSoWELL survey. Second, we validate the methodological basis of the present resource. The premise of the CoSoWELL project rests on the idea that the four story types—related to distant past, yesterday, future, and the Cookie Theft scene description control—elicit written productions that are formally and functionally separable as well as foreground distinct psychological facets of individuals’ lived experience (Brown, 2021). Moreover, we expect that linguistic patterns in CoSoWELL narratives reflect psychological changes related to the initial and continuing experience of the pandemic, which may additionally vary across story types. To achieve this second objective, we examined the effects of story type and the temporal measure of the test session, as well as their interaction, on linguistic structure and content of the narratives that constitute the CoSoWELL corpus. Below we outline five quantitative analyses employed to pursue the present objectives.

Analysis 1 focused on the lexico-syntactic formal properties of the narratives elicited under each story type to determine whether each story type forms a distinct linguistic profile (Labov & Waletzky, 1967). Beyond the comparative description of language patterns across story types, this analysis contributed to autobiographical memory studies which show a growing interest in incorporating linguistic variables as part of their analytical toolkit (e.g., Berna et al., 2016; Lempert, MacNear, Wolk, & Kable, 2020).

Remaining Analyses 2–5 zoomed in on the content of the narratives. Previous studies examining life stories have demonstrated that landmark events tend to be culturally shared regardless of individual differences in life trajectories (e.g., Brown, Shevell, & Rips, 1986; Brown, 1990; Brown & Lee, 2010; Brown, Schweickart, & Svob, 2016; Thomsen & Berntsen, 2008; Thomsen et al., 2011; Vanaken, Bijttebier, Fivush, & Hermans, 2021). Moreover, these shared experiences are reflected in the themes evoked during the writing process. Traditionally, this type of content analysis of life stories has been primarily carried out relying on a manual encoding schemata either for structural properties (for discussion about prior studies see Reese et al., 2011) or thematic analysis (e.g., Thomsen & Berntsen, 2008). However, this type of manual encoding of narratives does not scale to a one-million-word corpus. Additionally, a predefined schema may not be nimble enough to cover new themes that may have arisen for example due to the global pandemic.

To ensure an objective and scalable content analysis, Analysis 2 applied an unsupervised machine learning algorithm of structural topic model (STM) (Roberts, Stewart, & Tingley, 2019) to discover what themes and experiences emerge as identifiable topics from the corpus narratives and how they are distributed over the story types. Analysis 3 additionally examined whether a specific story type can be accurately identified (and thus functionally separated from other story types) based solely on the distribution of topics in the narratives belonging to this story type. Analysis 4 took the investigation one step further and explored semantics of the topics that are associated with different story types and compares these findings against the themes established in previous autobiographical memory studies. The final Analysis 5 tracked the temporal change in the frequency of specific topics across different story types and test sessions, before and during the first year of the COVID-19 pandemic. This analysis of the story type × test session interaction linked the dynamics in the content of the CoSoWELL narratives with the unfolding of the global lockdown (Brown, 2021).

The structure of the paper is as follows. The next section outlines the design and method of creating the CoSoWELL corpus, followed by the description of the data we make publicly available in this corpus release, along with descriptive statistics. Subsequent sections present quantitative analyses of the data, focusing first on the lexico-syntactic profiles of the story types and then transitioning into content analysis, which utilized topic modeling. The General discussion elaborates on the implications of our findings for psychological research and identifies several research questions that future work on CoSoWELL data can fruitfully address.

## Methods

### Participants

Participants at test sessions t1 and t2 were recruited through the web-based crowdsourcing platform Amazon Mechanical Turk (mturk.com) and those at t3–t5 through a different crowdsourcing platform, namely Prolific (prolific.co). Throughout the entire data collection, we used filtering options of the crowdsourcing platforms to only include individuals who were at least 55 years old, were born and also currently resided in Canada or the US, and were native speakers of English. Each participant in this project was offered an option to complete two separate online studies. One study contained a survey including questions pertaining to demographic information and psychological state, while another instructed participants to submit free-form written narratives following simple prompts (see details below). While most participants completed both studies, some opted for completing just one. Also, participants were able to take part in the narrative writing study at multiple test sessions, but they were only able to complete a single survey.

A total of 1502 unique participants submitted their responses to the CoSoWELL survey. Despite the use of filtering, a small fraction of participants did not meet the above-mentioned criteria. This release of the CoSoWELL makes available the survey data from 1451 participants aged 55 and older (age: *M* = 63.14, *SD* = 5.34; 946 female, 500 male, five others or prefer not to say). A largely overlapping but different set of 1178 unique participants took part in the narrative writing task. A total of 1028 participants (age: *M* = 62.88, *SD* = 5.29; 693 female, 332 male, three other or prefer not to say) have completed both the survey and the narrative writing tasks. In contrast to the survey task, the participants in the narrative writing task could contribute to one or more different test sessions. This method of data collection provides access to repeated behavioral records from multiple participants that enable both the cross-sectional and repeated-measures analyses. Summary information of the number of participants and the number of times they participated in different test sessions is provided in Table 2.

**Table 2:** Summary of participation in CoSoWELL tasks presented by the number of completed test sessions

	Number of test sessions			Total
CoSoWELL	1	2	3	
Survey	1451	0	0	1451
Corpus	687	315	176	1178
Survey and corpus	546	308	174	1028

This CoSoWELL release makes available both the complete set of the written narratives regardless of the availability of the participant’s survey data (*n* = 1178) and also the subset of the written narratives for which the participant’s survey data are available as well (*n* = 1028). This reporting enables researchers specifically interested in textual data to avoid data loss incurred by the unavailable survey data for a small fraction of participants (roughly 4.5%). We refer to this release of the written narratives as the CoSoWELL corpus (version 1.0). For the breakdown of sample sizes in the corpus data by age, see Table 3, by education level see Table 4 and by test session, see Table 5. We refer to the accompanying survey data as the CoSoWELL survey.

Participation was voluntary, and required participants’ informed consent prior to the beginning of the survey and the narrative writing task. Consent was also obtained for data usage; all participants reported here provided permission to use and publish their data. This study was approved by McMaster Research Ethics Board (ethics protocol #0606).

**Table 3:** Number of participants by the CoSoWELL components and age group

		Age (*n*)			
CoSoWELL	*N*	55–59	60–64	65–69	70–100
Survey	1451	536	473	297	145
Survey and corpus	1028	409	315	208	96

The education levels of the participants are reported in Table 4. The levels of education were collapsed from seven to four (high school diploma or lower; partial or complete college education; bachelor’s university degree; graduate degree).

**Table 4:** Number of participants by the source of the data in CoSoWELL and level of education including missing data (NA)

		Level of education (*n*)				
CoSoWELL	*N*	High school or less	College (complete or partial)	Bachelor’s	Graduate degree	NA
Survey	1451	134	530	488	286	13
Survey and corpus	1028	86	372	336	229	5

### Materials

#### Narrative writing task

This task was not time-limited but completion time was recorded as part of the study. Prior to the narrative writing task, the participants were instructed to avoid providing information that could be used to directly identify them, such as personal names. The four stories were written separately in a sequence. For writing a given a narrative, a large text box was provided in a web browser with a prompt above providing the instructions about the type of story. All the participants received the same instructions and the stories were written in the same order. The instructions for the story types are provided below and the order is indicated with the number given in bold (but it was not part of the instructions).

- **Story 1:** Write a story about a significant life event that occurred in your distant past.
- **Story 2:** Write a story about a personal life event that occurred yesterday.
- **Story 3:** Write a story about a personal life event that will take place in your future.
- **Story 4:** Write a story about the event described in the picture.

We labeled the resulting story types as “past”, “yesterday”, “future”, and “cookie”.

#### Social and cognitive well-being survey

As part of the CoSoWELL corpus, a survey was administered that included questions related to a social and cognitive well-being as well as demographic information. The present CoSoWELL release (version 1.0) makes available 39 variables pertaining to educational, social, and cognitive characteristics of individual participants. The complete list of the questions and their associated summary statistics, when applicable, is provided in the Supplementary materials (supplementary_material_cosowell). Several sections of the survey will be processed and reported with future releases of the CoSoWELL project. We remind the reader that the focus of the present study is on the corpus data, while the comprehensive analysis of the CoSoWELL survey data is relegated to future work (which can be conducted by the research community that receives access to the data with this publication).

### Procedure

Participants in the narrative writing task first read the letter of information and provided their consent by pressing the Continue button. They were then presented with the instructions (see above) and typed their narratives in the text boxes. The task was untimed and there was no limit on the length of the response. On average, the participants spent 34 (*SD* = 24) min completing the narrative writing task.

For participants in t1 and t2 sessions who were recruited via Amazon Mechanical Turk, the narrative writing task was programmed in the platform’s web interface. In test sessions t3–t5, participants were recruited via the crowdsourcing platform Prolific: these participants were directed to the LimeSurvey online platform where the writing task was implemented. Typed responses were recorded when participants completed the task by pressing the Submit button. Participants were compensated for the time by the payment of 7 USD.

Upon completion of the task, all participants were invited to complete an online survey that included questions about social and cognitive well-being as well as demographic information. This invitation was sent out roughly 1 week after completing the narrative writing task. The completion of the survey took 10–15 min. Participants were compensated for their time by the payment of 2 USD. The survey was administered using the same crowdsourcing platform and the same data collection software as the writing task (Amazon Mechanical Turk for t1 and t2, and Prolific and LimeSurvey for t3–t5).

### Variables

The goal of this paper is two-fold: 1) the description of the creation and release of the data collected in the CoSoWELL project and 2) the validation of the written narrative task as a data collection method for the purposes of investigating aspects of autobiographical memory. Thus, only a selected set of variables pertaining to the survey data are covered in this study. One critical independent variable of the present paper was the categorical variable story type coded with four levels: “past”, “yesterday”, “future”, and “cookie”, corresponding to the four thematic prompts of the narrative writing task cited above. As outlined in the Introduction, the premise of this data collection effort was that the four story types would manifest differences in the linguistic structure and content and highlight different aspects of autobiographic memory.

An additional independent variable of interest was the variable test session, a categorical variable with t1–t5 as levels. This variable taps into a temporal unfolding of the change in written productions from the pre-pandemic state (t1) throughout the first year of the pandemic (t2–t5). Since we expected the psychological impact of the unfolding pandemic to differentially affect one’s perception of the distant past, the recent past and one’s future outlook, we considered the interaction between story type and test session along with the main effects of the interaction terms.

## Results

Analyses below make use of the corpus of narratives produced by 1028 participants who also completed the CoSoWELL survey. This dataset contains 1,223,033 tokens or 91% of the data in the full CoSoWELL corpus (which includes participants who have not completed the survey).

The remainder of this section has the following structure. First, we report descriptive statistics of the entire corpus and specific test sessions. Second, we outline Analysis 1 of eight linguistic variables representing lexical and syntactic features of the written texts by story type. Third, we present a series of Analyses 2–5 based on the structural topic model, which was fitted to the words and narratives comprising the corpus and identified—in a programmatic and unsupervised way—the topical structure of those narratives. All the analyses carried out in this study were conducted in R version 3.6.3 (R Core Team, 2020).

### Preprocessing and descriptive statistics of CoSoWELL

The collected narratives were tokenized, tagged for part-of-speech (POS) and morphological information, lemmatized, and syntactically parsed using the UDpipe version 2.0 implemented in the R package udpipe, version 0.8.5 (Straka & Straková, 2017) with a pretrained English model, version 2.5-191206 (additional information is provided at https://ufal.mff.cuni.cz/udpipe). In terms of POS, the schemas based on universal POS as well as that based on the Stanford POS are both included in this corpus release. In the released data, the former is labeled as upos and the latter as xpos.

The syntactic model follows the Universal Dependency schema (UD) (Nivre, 2015), version 2.4. This version of the UD schema is available for 83 languages providing opportunities for carrying out linguistically meaningful typological analysis at scale (for discussion see Croft, 2017; Croft, Nordquist, Looney, & Regan, 2017). Additional information about this schema is also provided online at https://universaldependencies.org/format.html.

**Fig. 1:**
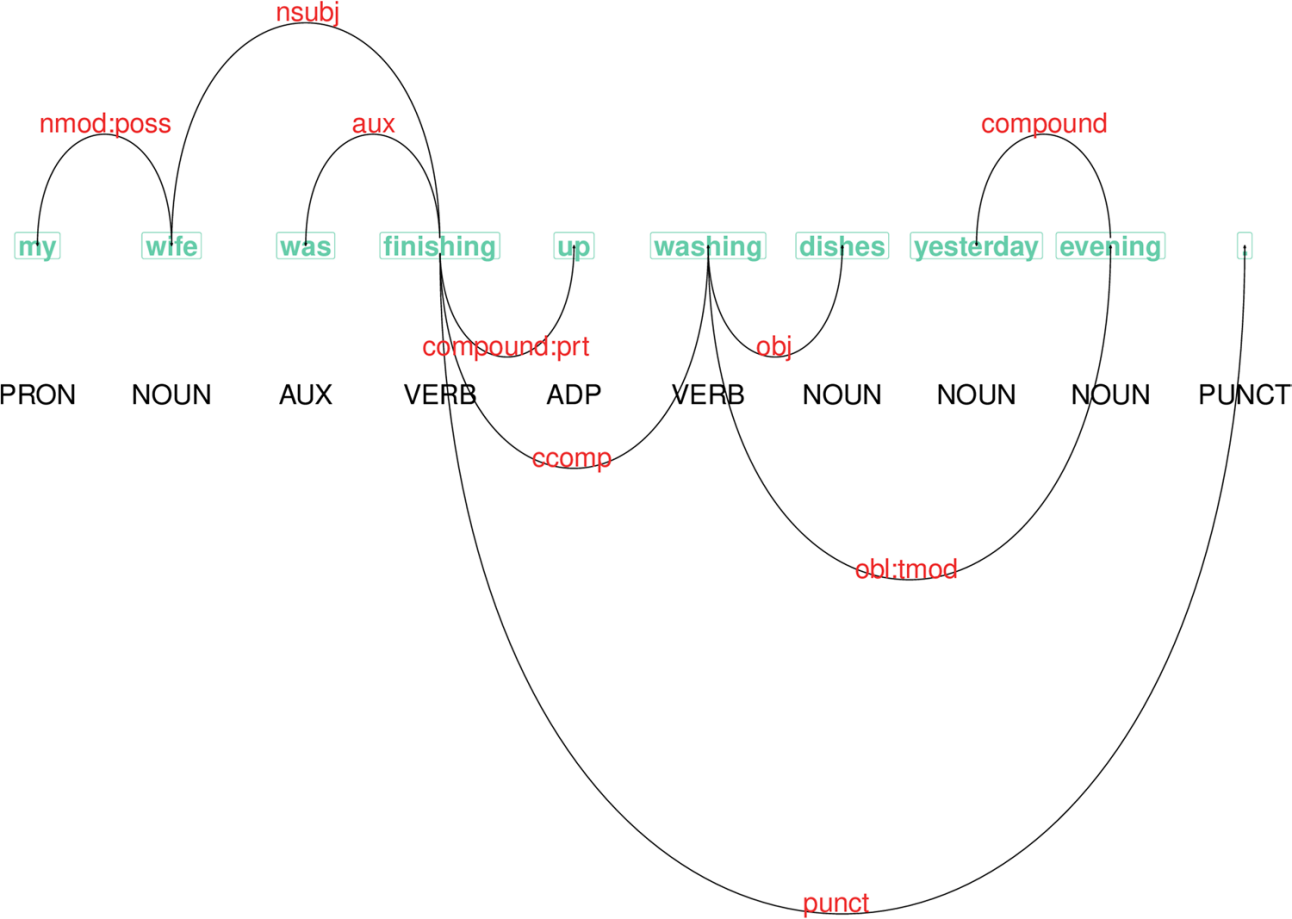
Visualization of the output of the preprocessing pipeline for one sentence. The *arcs* represent dependency relations. Tokenization is given in *green*, part-of-speech tagging in *black* and the labels of the dependency relations in *red*

The UD schema is based on the shared premise of dependency-based grammars in which syntactic structures are (primarily) assumed to consist of binary relations between words. The relation is asymmetric, where one word is a dependent of another word referred to as the head. This allows the UD schema to represent dependency relations among words as a tree structure that emphasizes the functional characteristics of the relations (for recent discussion and overview see de Marneffe & Nivre, 2019). Figure 1 illustrates the output of the preprocessing pipeline of the narratives with the sentence: *My wife was finishing up washing dishes yesterday evening.*. The dependency relations are drawn as arcs over the sequence of words starting from the root, i.e., *finishing*.

Corpus size can be described in several linguistically meaningful ways. Tokens refer to the smallest individual units of which a corpus is comprised. Here, tokens include words, numbers, symbols, and punctuation. Types reflect the total number of unique tokens present in the data: thus, any number of occurrences of a token *dog* would count as one word type. A lemma (or “word stem”) is the base form of a word, representing its simplest morphological realization (e.g., the lemma for *dogs* and *dog* is *dog* and the lemma for *baked* and *baking* is *bake*). An English lemma is also the citation form of a word provided in a dictionary. Thus, the category lemma captures the number of unique lemmas in the corpus.

In Table 5, we report descriptive statistics for the two versions of the CoSoWELL corpus: 1) the complete CoSoWELL corpus representing all participants as well as its subset, which contains the written narratives for the participants who also completed the CoSoWELL survey. The descriptive statistics are broken down by the specific test sessions: t1 = 2019; t2 = from 2020-04-08 to 2020-06-16; t3 = from 2020-06-17 to 2020-06-30; t4 = from 2020-10-14 to 2020-11-05 and t5 = from 2021-01-12 to 2021-02-15.

**Table 5:** Descriptive statistics of the corpus by the test session. The upper part summarizes corpus data from all participants in the narrative writing task. The lower part summarizes data from the participants who completed both the narrative writing task and the survey (corpus + survey)

Test session	Token	Type	Lemma	Narrative	Sentence	Participant
	CoSoWELL corpus					
t1	161,181	9462	7787	848	9278	212
t2	397,416	14,670	12,195	2111	22,497	402
t3	270,443	12,779	10,571	1708	15,137	427
t4	248,931	12,058	10,002	1640	13,588	409
t5	260,349	12,461	10,318	1668	14,315	395
Total	1,338,320	61,430	50,873	7975	74,815	1845^∗^ (1178)
	CoSoWELL corpus + survey					
t1	158,656	9394	7728	832	9127	208
t2	326,118	13,252	10,978	1727	18,429	317
t3	256,606	12,427	10,272	1604	14,371	401
t4	224,696	11,407	9483	1480	12,274	370
t5	256,957	12,378	10,251	1640	14,105	388
Total	1,223,033	58,858	48,712	7283	68,306	1684^∗^ (1028)

The number marked by ^∗^ represents the total number of participants across test sessions, counting participation in each test session separately. The *number in parentheses* represents the unique number of participants across all test sessions.

The released CoSoWELL corpus (version 1.0) follows the “tidy” data format where each variable is a column, each observation is a row and each type of observational unit is a table (Wickham & et al. 2014). In terms of text data, the format of the tidy data corresponds to representing the processed text as a table with one-token-per-row, i.e., long format. While this format increases the resulting file size, the analysis of the text data becomes significantly more convenient, especially when combined with the survey data.

### Analysis 1: Lexico-syntactic profile and story type

In this section, we explore linguistic variation across story types, with a focus on lexico-syntactic variables. The overarching goal of this analysis is to determine whether differences in story types—presumably related to different facets of autobiographical memories and ideation—manifest themselves in formal linguistic properties of produced stories. A total of eight variables were explored, many of which are in common use in computational-linguistic analysis of text and lexico-syntactic complexity and structure (Baayen, 2001; Graesser, McNamara, & Kulikowich, 2011; Jagaiah, Olinghouse, & Kearns, 2020; Kyle, 2019; Nippold, Cramond, & Hayward-Mayhew, 2014; Vermeer, 2000).

These variables included noun-to-verb ratio where higher values may signal telegraphic style characteristic of cognitive impairment; an additional set of three variables tapping into lexical diversity of produced narratives (different operationalizations of type-token-ratio); another set of three variables tapping into syntactic complexity of each sentence, further aggregated by story (e.g., the number of syntactic dependencies normed by sentence length, the maximum depth of the syntactic tree, and the proportion of constituent phrases in the sentence); and finally mean length of (written) utterance as a measure of writing ability. For motivation and references, see Supplementary materials S1. Regression models fitted to each of the eight dependent variables demonstrated a strong effect of story type. These findings suggest that the story types are structurally separable based on their lexico-syntactic form, in line with our expectation. For full details of analyses, see Supplementary materials S1.

### Topic modeling

Topic modeling is a primary tool of our analyses. We used it to identify the content of narratives elicited under different instructions and thus associated with different story types; quantify differences in content between the story types; link the content of specific story types with the distinct facets of autobiographical memories that they tap into; and finally determine the general and specific changes in the content of story types over the period of data collection, prior to and during the COVID-19 pandemic.

Topic modeling belongs to the family of unsupervised machine learning algorithms, i.e., methods used to discover underlying structure in unseen data based solely on those data, without resorting to the researcher’s intuition. Topic models operate on distributions of word frequencies within and across documents and estimate a data generating process for each document. The generative language component of the model starts with two distributions: one associates documents with topics and another associates topics with words used in the documents (Blei, Ng, & Jordan, 2003; Roberts, Stewart, & Airoldi, 2016; Roberts et al., 2019); for an elaboration of the data-generating process see Supplementary Materials S2.

After training, a topic is defined as a mixture of words that often co-occur with each other within and across documents in the given text base. Furthermore, each word in the available data is associated with a probability of pertaining to a specific topic. Words with a high probability of being connected to a given topic are the most salient representatives of this topic relative to other topics: Such words are used by researchers to provide a post hoc label for the topic and determine its content. Similarly, a trained topic model represents a document as a mixture of topics, with some topics typically represented in the document more saliently (with a higher probability) than others (Blei et al., 2003; Roberts et al., 2019). Thus, a document about a single event such as wedding may represent several themes, e.g., people’s names and social roles (e.g., bride, groom, best man, mother-in-law), location, time, food, and others. Topic modeling allows for an objective determination of recurring latent language patterns—topics—in text data and makes it possible to identify multiple topics that contribute to a given narrative.

Most often, topic models are used for automatic categorization of documents based on shared topics, which makes topic modeling indispensable for programmatic creation of data archives, e.g., bioinformatics (for an overview see Liu, Tang, Dong, Yao, & Zhou, 2016) and social sciences (for an overview see Roberts et al., 2016). In psychological research, topic modeling has largely been used in one of two complementary ways: to objectively identify the most common ideas or themes recurring in the published literature (Hall, Jurafsky, & Manning, 2008; Kuperman, Jarema, & Libben, 2021; Priva & Austerweil, 2015)or use the estimated topics as features (see below). Previous studies have demonstrated that the estimated document-topic distribution can be used in a supervised setting, for example to predict writers’ personality and gender in (Schwartz et al., 2013), state of depression of the writers in (Eichstaedt et al., 2018) or the emotional state in (Sun, Schwartz, Son, Kern, & Vazire, 2020). In this study, we utilized topic modeling in both ways. First, the study supplied the narratives of the CoSoWELL corpus as the input for topic modeling in order to discover latent topics. Additionally, we utilized document-topic distributions as features in modeling the differences between the story types in Analysis 3. As part of the data release, the outcomes of the trained topic model are made available for future use.

The standard algorithm underlying topic modeling is the latent Dirichlet allocation (Blei et al., 2003). In this study, we utilized a more sophisticated structural topic model (STM) developed by (Roberts, Stewart, Tingley, & Airoldi, 2013). The main advantage of STM is in its ability to incorporate meta-data pertaining to the documents (e.g., age or gender of the writer, date of test session, or story type) as covariates that contribute to the allocation of words to topics and topics to documents. In other words, STM can be used to estimate latent topics for specific story types or test sessions or even the interaction between the two factors. We made use of this option in Analysis 5. However, the initial goal of our content analysis was to assess whether the topics associated with the narratives in the corpus could be used to discriminate between the story types even when the machine learning algorithm had no access to information pertaining to the story types. If we can demonstrate that narratives were associated with different topics and that these topics could be used to accurately classify the narratives into the four story types, this would prove the validity of our design decision, i.e., the use of the elicitation prompts to tap into distinct facets of autobiographical memories. For this reasons, Analyses 2–4 relied on the basic STM algorithm that discovers topical structure from text documents (elicited narratives in our case) without factoring in any covariates.

#### Model fitting

We used lemmas rather than tokens (see definition of lemma above) in the subsequent modeling, which alleviates data sparsity issues. To ensure that sufficient data were available, all stories shorter than three sentences were removed from the analysis. Additionally, all lemmas that had a frequency less than two and did not appear at least in two narratives were removed from the data. Finally, all lemmas listed in the English stop word list provided in the R package quanteda were pruned from the data (Benoit et al., 2018). The resulting data set consisted of 6816 narratives and 11,261 lemmas serving as the vocabulary in the STM.

To estimate an optimal STM given the data, we searched for the number of topics that provides the best semantic quality of the fitted model: This number is labeled as the hyperparameter *k* and is not known a priori. We fitted models while ranging *k* from 3 to 30 and recording values of semantic coherence and exclusivity for each model. Semantic coherence proposed by (Mimno, Wallach, Talley, Leenders, & McCallum, 2011) is designed to measure semantic quality of the topics. The score is similar to pointwise mutual information and reaches its maximum value when the most probable words in a topic also have a high frequency of co-occurrence with each other. While semantic coherence strongly correlates with expert judgements about the quality of topics (see (Mimno et al., 2011)), this score has been shown to provide inflated estimates when a small number of topics is dominated by frequent words (Roberts et al., 2014). Thus, following (Roberts et al., 2019) we combined coherence with another measure, namely exclusivity. This measure of semantic quality factors in both word frequency and the degree to which it is particular to a specific topic. The R package STM provides an implementation of this score referred to as FREX (Airoldi & Bischof, 2016; Bischof & Airoldi, 2012).

**Fig. 2:**
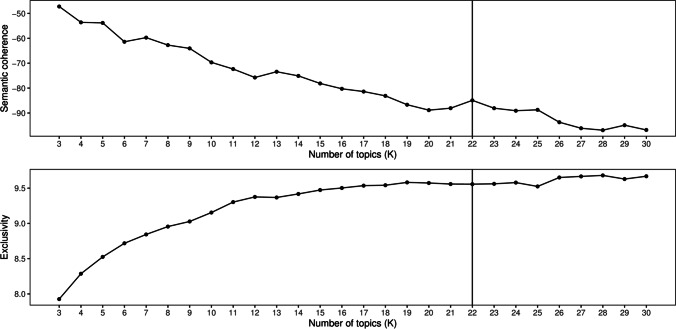
Model diagnostics for evaluating the number of topics. The *vertical line* indicates the chosen model (*k* = 22)

The results of the search for the hyperparameter *k* are visualized in Fig. 2. For the purposes of the present study, we chose the model with 22 topics as it represented a balance across the two indices, i.e., a high value of semantic coherence and exclusivity (for discussion see (Roberts et al., 2019)). Specifically, semantic coherence had a small peak at this K while the respective value of exclusivity was located in a plateau, i.e., was identical to the values found for 20–25 topics. For consistency with the observed advantage in semantic coherence, we settled on 22 topics. This final model was further supported by our qualitative analysis of the topics where we carried out a close reading of the narratives along with their associated topics. Thus, the data were refitted with *k* set to 22. We will refer to this final model simply as the final STM. Analyses 2–4 reported below are based on this model.

#### Interpreting topics through keywords

**Fig. 3:**
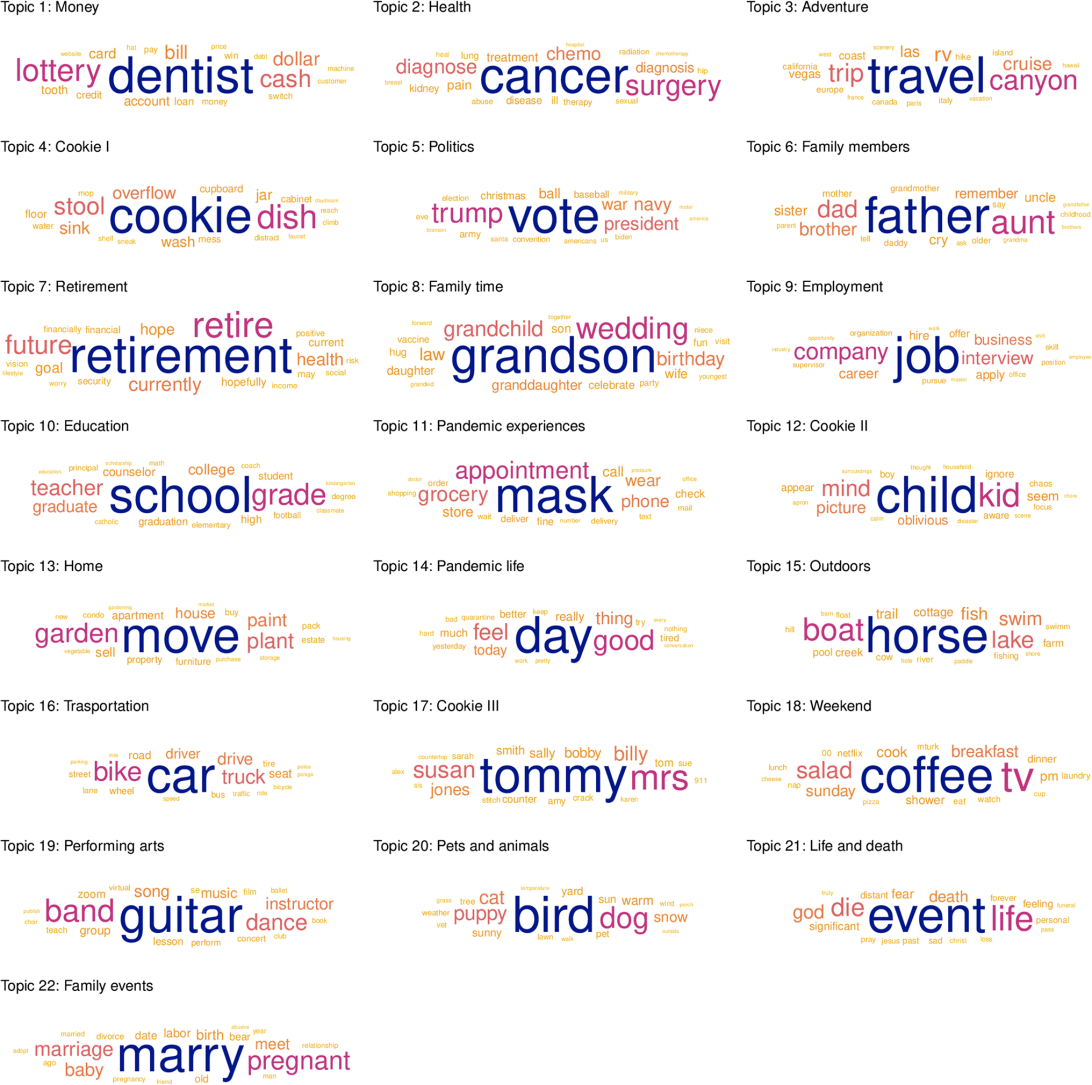
A word cloud of the estimated keywords for each topic. The most important keyword of a given topic is placed in the center of the cloud. The keywords are presented in rank order and the ranking is further denoted with the size and color

To gain a better understanding of the content of the estimated topics in the final STM, we extracted 20 keywords for each of the 22 topics using the FREX method. The resulting keywords are visualized as a word cloud in Fig. 3 and also provided as a separate file for use in future studies. Additionally, we assigned a label for each topic. It is important to emphasize that the label is a researcher’s subjective summary of a particular topic’s content rather than its objective or authoritative interpretation. It is also important to remember that the number assigned to a particular topic is arbitrary.

In general, the keywords associated with a particular topic indicated that STM was able to recover latent topics from the narratives that cognitive aging research has previously identified as recurring themes in manually coded life stories (e.g., Thomsen & Berntsen, 2008; Thomsen et al., 2011). For example, (Thomsen et al., 2011) reported 22 manually coded themes (coincidentally matching with the number of topics in our study) that covered a broad range of life circumstances associated with a personal life story. These themes ranged from childhood and education to such aspects of adult life as work, family and spare time, to older age such as grandchildren and retirement to death and illness (see also Kenyon et al., 1999). Family life also figured prominently in the topics estimated on the basis of the current corpus. Older adults produced life stories with elements from their personal experiences from family time to family events as well as with a generational perspective (family members) (Randall et al., 2004). A common theme of work reported in prior literature resonated with topics like money and employment. At the same time, some of the estimated topics have not figured prominently in previous studies, including politics and, obviously, experiences related to the global pandemic, namely pandemic experiences and pandemic life. The ability of the computational method to identify topics of relevance in the absence of a fixed scheme showcases the strength of this method.

Since we used the Cookie Theft picture as a standard baseline for eliciting stories, we expected the resulting narratives on this topic to stand out in comparison to the three other story types. Indeed, this appeared to be case as three of the estimated topics were directly related to story type “cookie”. We will refer to these simply as cookie I, cookie II, and cookie III. Interestingly, these topics were functionally separated. The topic cookie I was estimated to pertain to describing the objects in the picture. This is important as the inability to describe the salient objects in the picture has been connected to language impairments with advancing age (for discussion see Cummings, 2019). The topic cookie II focused on verbalization of the child as part of the scene depicted in the picture and the keywords in the third topic emphasized verbalization of other depicted characters and specifically the act of naming them.

Given the circumstances of the data collection, i.e., COVID-19, it is not surprising that two topics directly referring to the pandemic emerged from the life stories, namely pandemic experiences and pandemic life. The topic pandemic experiences appears to capture episodic memories of everyday events and how these have been influenced by the COVID-19 pandemic, with such keywords as *mask*, *appointment*, and *grocery*. On the other hand, the topic pandemic life pertains to the perception and appraisal of one’s daily life during COVID-19, with keywords like *feel*, *quarantine*, *good*, *bad*, and *tired*.

With the topic model described above, the remainder of the Results section examines the validity of the CoSoWELL corpus as a research tool by reporting targeted analyses of the topics against story types and time.

### Analysis 2: Topic distributions and story types

Thus far the analysis of the topics generated by the model has demonstrated that they were linguistically meaningful. However, this type of content analysis alone does not inform us whether the story types used as a prompt are functionally separable. This is critical for the purposes of the present study as the premise of the study relies on the fact that the cookie story served as a control and the other three story types (past, yesterday and future) evoked different autobiographical memories. It is important to remember that the fitted topic model was unsupervised and it was never exposed to the information pertaining to the story type during training. Thus, any detectable signal pertaining to the story type emerged directly from the learning process. We turn to document-topic distribution to further investigate the relationship between the elicited life stories and autobiographical memory.

**Fig. 4:**
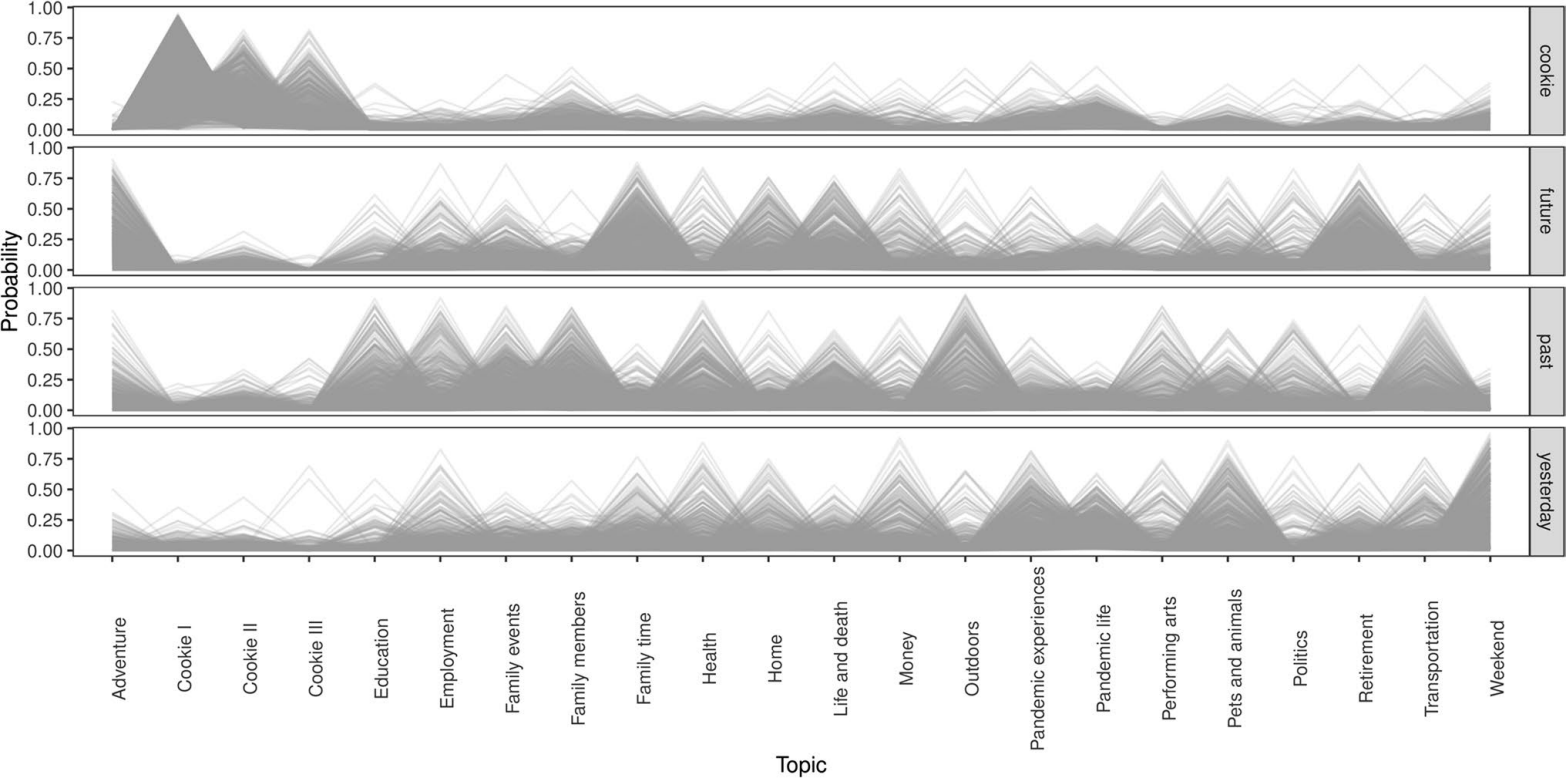
Estimated document-topic probability distribution across the four story types. Each *shaded line* represents a life story and its document-topic probability distribution

A topic model associates a given narrative with a specific document-topic distribution: Here, each narrative comes with an estimated probability distribution across the 22 topics. The document-topic distribution depicts which topics were estimated to be more prominent in a given narrative. The document-topic distributions open a window into the content evoked by the story types (past, yesterday, future and cookie) and then their subsequent verbalization. By way of example, in Supplementary materials S3, we illustrate the property of a topic model to associate a specific narrative with a document-topic distribution by considering two extreme cases: a narrative that was estimated to be dominated by a single topic while the second story was estimated to display multiple topics simultaneously. The overall document-topic probability distribution of the narratives relative to the four story types is visualized in Fig. 4.

It is evident that certain topics were predominantly associated with a specific story type, see below. For instance, the document-topic probability distribution for the story type depicting the Cookie Theft featured three peaks in the overall distribution, not found in any other story type. Thus, this story type demonstrated a clear-cut and expected separation from the other three story types. Similarly, the topic labeled Outdoors was strongly present in the narratives about distant past, but not those about the recent past or the future, which congruent with the pandemic-related isolation. Retirement, on the other hand, was a strongly prominent topic for the Future narratives, but virtually absent from other story types. This, again, is congruent with the large proportion of pre-retirement individuals among the CoSoWELL participants. Overall, the visualization of the document-topic probability distribution indicated a degree of alignment between constellations of specific topics and particular story types although the final STM was not aware of the story type during the model fitting. In the following analysis, we formally test this separability of the story types based on the document-topic probability distribution.

### Analysis 3: Predicting the story type of a life story

This analysis used the computational technique of random forests to classify the narratives into the story types based on the document-topic distribution that the final STM estimated for each narrative. As a reminder, the fitted STM has never been exposed to the story type information during its learning process. If the topical structure of a narrative (encoded in the document-topic distribution, Analysis 2) can accurately predict its story type, this demonstration would give a positive answer to the critical question. It would demonstrate that the prompts used for the story types were successful in eliciting structurally (Analysis 1) and functionally distinct and semantically identifiable linguistic patterns in participants’ narratives.

#### Model fitting of the random forests

To investigate the separability between the four story type, we used a random forests technique (RF) implemented in the R package ranger, version 0.12.1 (Wright & Ziegler, 2017), a more efficient implementation of the original algorithm by (Breiman, 2001). This technique is widely used in language research, including reading (e.g., Matsuki, Kuperman, & Van Dyke, 2016), phonetics (Arnhold & Kyröläinen, 2017) and language variation (e.g., Tagliamonte & Baayen, 2012) among others. We used the same data set across the RF and STM. However, before training the RF, the data were split into training (75%) and testing (25%) with stratified sampling to ensure that the critical variable of story type was balanced across the splits of the data. The RF were used to classify the four story types as a function of the 22 document-topic distributions.

There are effectively two hyperparameters that are generally considered to impact the performance of the RF, namely *mtry* controlling the number of predictors that are randomly sampled and available during a split in a given tree and *num.tree* controlling the number of the trees used in the model (Breiman, 2001; Strobl, Malley, & Tutz, 2009). A grid-search was carried out using a fivefold cross-validation based on the training data to find the optimal value for them. In the grid search, the *mtry* was set to 2, 4, 6, and 8 (by default this hyperparameter is set to $\sqrt {p}$ where *p* is the number of predictors used in the modeling). The *num.tree*, was set to 500, 1000, and 2000. Area under the ROC curve (AUC) was used as the optimization criterion. The results of the tuning process showed that the optimal values for the hyperparameters were: *mtry* = 6 and *num.tree* = 1500. Lastly, the RF were retrained on the full training data using these tuned values for the hyperparameters. All other parameters of the model were kept at their default value. The evaluation of the model performance is reported in the next section.

#### Model evaluation of the random forests

The RF achieved an excellent classification performance on the test data with an AUC of .96 (Hand & Till, 2001). Critically, the balanced accuracy of predicting a story type for a given narrative based on the document-topic distribution for that narrative was 89%. The predictive performance of RF on the test data is reported as a confusion matrix in Table 6.

**Table 6:** Confusion matrix of the RF based on the test data. Correctly classified narratives are located on the diagonal

	Observed			
Predicted	cookie	future	past	yesterday
cookie	402	0	3	3
future	2	332	43	59
past	9	29	366	49
yesterday	3	55	31	316

The results demonstrated that the document-topic distribution learned by the STM captured meaningful linguistic patterns, which provide highly accurate classification into the four story types. This analysis supplies quantitative evidence to the successful discrimination between the story types, which was clearly visible in Fig. 4. That is, the prompts used for eliciting written production in the CoSoWELL project were effective in generating differences in verbalization of respective experiences. Additionally, “cookie” as a story type clearly functioned as a separate category from the other three. Only six stories of the “cookie” type were misclassified and as a story type it did not strongly attract any other types either. Hence, we can conclude that the use of the Cookie Theft picture fulfilled its primary function of serving as a control. At the same time, the important result here is the fact that the narratives themselves contained enough systematic linguistic information that could be harnessed and utilized in further analyses.

In sum, we have provided evidence that the methodology devised in this project fulfilled one of its core functions of eliciting linguistically distinct narratives. The prompts used in this study were sufficient to evoke different responses in the verbalization of the writers’ experiences. However, it is entirely another question whether the systematic nature of the story types also reflected aspects of autobiographical memory. The following section addresses this question.

### Analysis 4: Story types and themes of autobiographical memories

We asked whether the systematic patterns discriminating the story types mirror any aspects of autobiographical memory. To investigate this, we turned to the latent topics identified as relevant by STM and examined their contribution to the accuracy with which the random forests model classified narratives into story types.

As a first step, we estimated the relative variable importance for the 22 topics as predictors in the random forests classification model. This provided a global relative ranking of the topics in discriminating the four story types, highlighting the central role of both the autobiographical and pandemic-related ideation in the content of the narratives. The results are reported in Supplementary materials S4.

The global relative variable importance does not inform us about the contribution of a specific topic in discriminating a given story type. For example, the topic cookie 1 was ranked as the most important predictor by the RF model. While valuable, it does not provide information regarding which of the story types were discriminated by this topic or how strongly. This information is required in order to relate the narratives to aspects of autobiographical memory. Thus, as a second step, we evaluated the contributions of the individual topics in discriminating between the four story types. This was achieved by constructing a partial-dependence profile (PD profile) for each of the predictors (Friedman, 2001). This profile displays how the predicted probability for a specific story type varied given the values of a predictor while the other predictors were held constant. As there were four story types, a given DP-profile consisted of four estimates for a given predictor. In the case of cookie 1, this method provided an estimation of how the predicted probability varied for each of the story type when the values of cookie 1 changed. Hence, PD profile captures how the expected model prediction behaved as a function of a given predictor (topic) for each story type. We used the implementation in the R package DALEX, version 2.2.0, (Biecek, 2018). Here, we present the results for those topics that were estimated to have a strong positive correlation with a particular story type, i.e., the predicted probability for a specific story type increased as the values of the predictor increased.

**Fig. 5:**
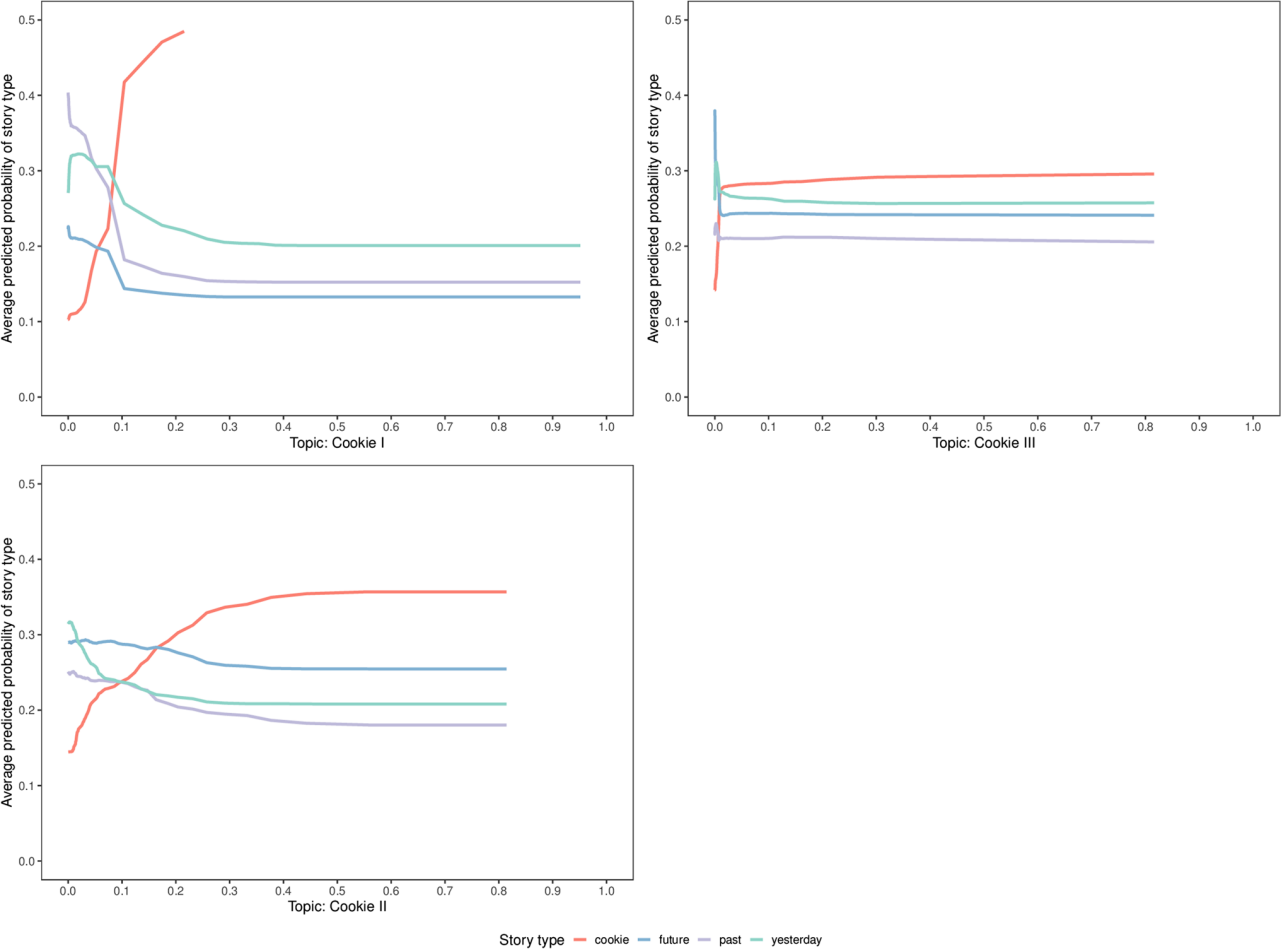
Estimated PD profiles of the most discriminative predictors for the story type “cookie”. The average predicted probability is given on the *y*-axis and the *x*-axis gives the values associated with a specific predictor (topic) broken down by the four story types

The PD profiles for the story type “cookie” are visualized in Fig. 5. As expected, the predictors that were labeled as cookie-related topics also served to discriminate the “cookie” story type from the other types. As the probability of the cookie-related topics increased so did the average predicted probability of the model for the “cookie” type.

**Fig. 6:**
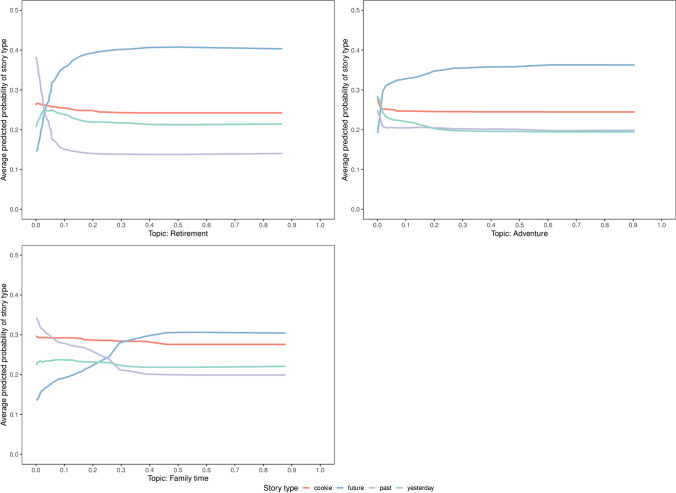
Estimated PD profiles of the most discriminative predictors for the story type “future”. The average predicted probability is given on the *y*-axis and the *x*-axis gives the values associated with a specific predictor (topic) broken down by the four story types

Story type “future” featured topics of retirement, adventure and family time as the strongest predictors. The PD-profiles are visualized in Fig. 6. The results indicated that the future outlook was strongly connected to episodic events, i.e., memories of specific, personally experienced events, such as the topic of adventure and family time (in line with Rathbone et al., 2015). Furthermore, retirement constitutes an important landmark event that marks a transition in the personal life story, as indicated in prior literature (see Thomsen & Berntsen, 2008). This aligns well with the strong positive correlation between the topic retirement and the future outlook.

**Fig. 7:**
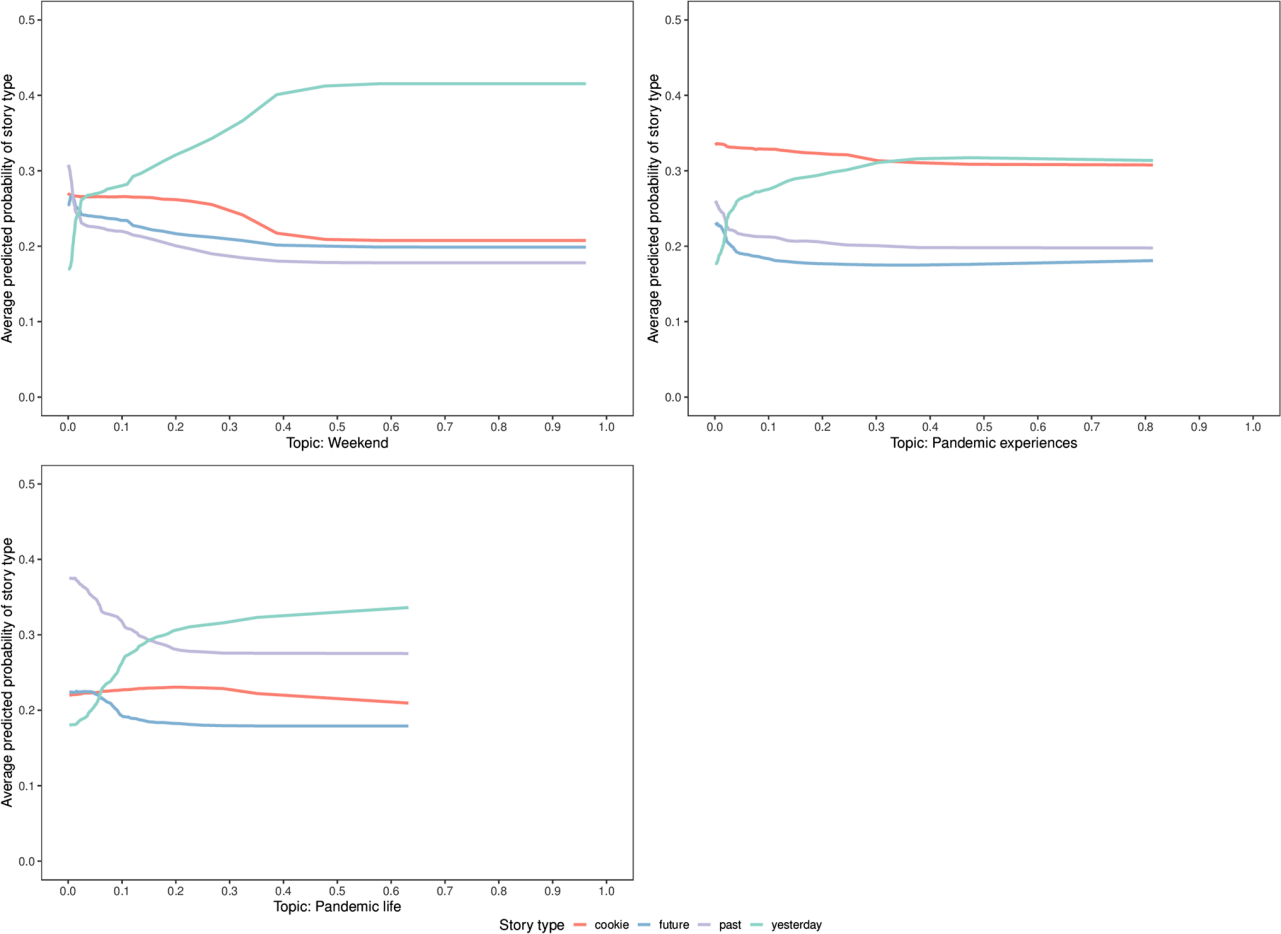
Estimated PD profiles of the most discriminative predictors for the story type “yesterday”. The average predicted probability is given on the *y*-axis and the *x*-axis gives the values associated with a specific predictor (topic) broken down by the four story types

The third story type examined here was “yesterday”. The contributions of the most important predictors are visualized in Fig. 7. All these topics indicated a strong link to episodic events, e.g., the topic weekend and the topics directly related to COVID-19, namely pandemic experiences and pandemic life. This finding points to the central role that the lived experience of the pandemic plays in the perception of the recent events.

Story type “past” was strongly correlated with the topics linked to family life, i.e., family members and family events displaying both semantic and episodic components. The critical role of these two topics was anticipated given prior research on autobiographical memories of personal life events in the past. They tend to be strongly influenced by family ties and the social activities that come along with these ties (Nelson, 1996). It is by telling and sharing these stories that tellers shape the constituent parts of their life stories (Fivush, 2008). The final predictor strongly association with the story type “past” was topic education. This also aligns with the prominent role of education as a formative experience that has emerged in results of previous studies on autobiographical memory regarding past life events (e.g., (Thomsen et al., 2011)).

**Fig. 8:**
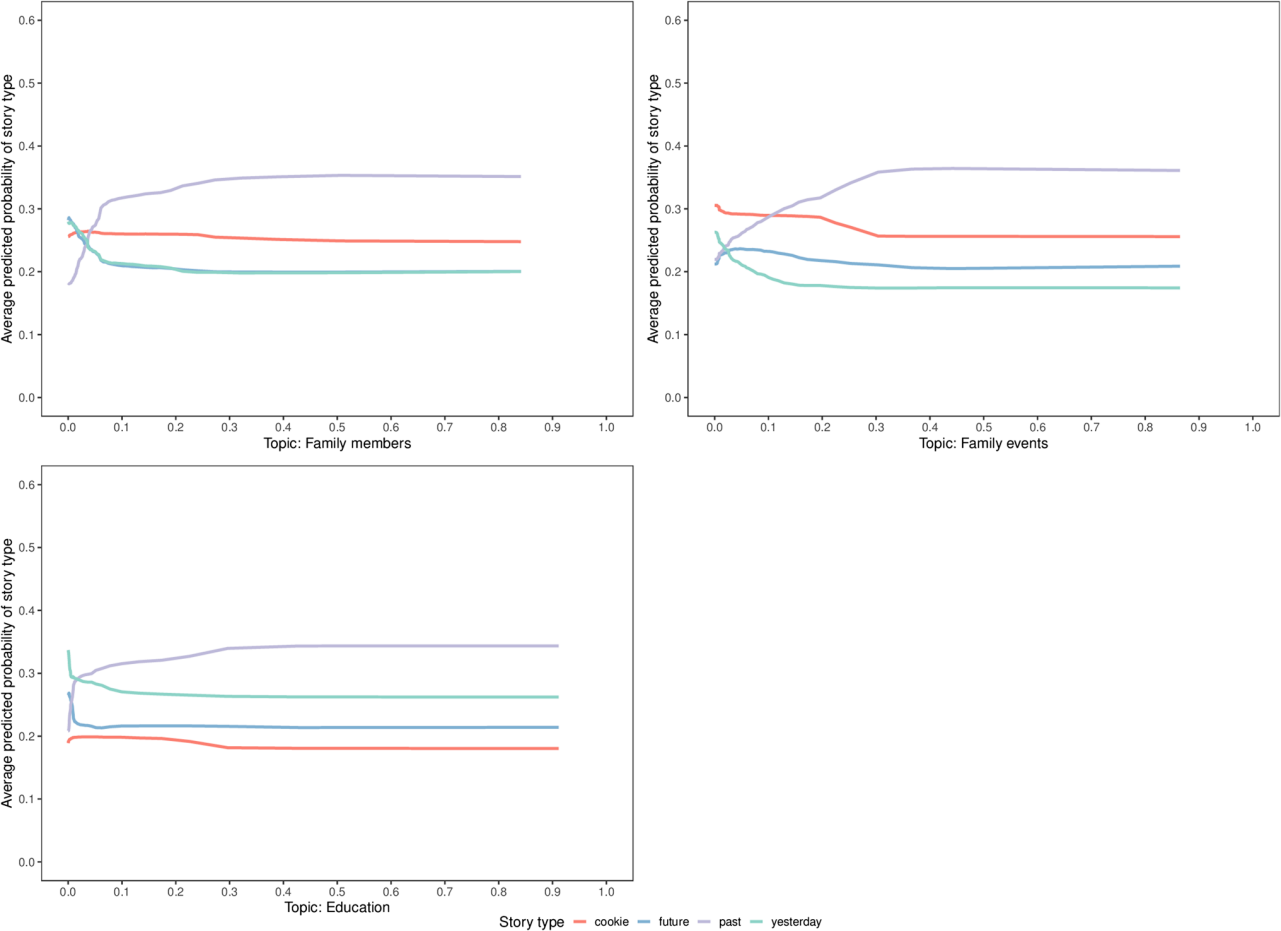
Estimated PD profiles of the most discriminative predictors for the story type “past”. The average predicted probability is given on the *y*-axis and the *x*-axis gives the values associated with a specific predictor (topic) broken down by the four story types

Analyses 3 and 4 jointly demonstrated that the document-topic distributions of individual narratives were strongly associated with specific story types. This suggests that the four story types produced markedly different responses in the participants. Moreover, the analyses showed that the topics that were estimated to provide the strongest discrimination between the story types were also aligned with previous studies investigating aspects of autobiographical memory. Thus, the collected narratives in the CoSoWELL corpus can be interpreted as faithfully reflecting aspects of autobiographical memory(Fig. 8).

### Analysis 5: Topic frequency changes over time

Analyses above established the number and nature of the topics that best represent the content of the CoSoWELL narratives across story types, yet did not address the temporal structure of this time-series. Given the dynamic context of the pandemic, it is expected that the relative frequency of some of the topics fluctuated in narratives produced at different time points. We harnessed additional functions of structural topic modeling (STM) that allow a formal estimation of the effect that covariates—like time and story type—may have on the allocation of words to topics and topics to documents. Specifically, we examined for each story type which of the 22 topics demonstrate a significant temporal change in frequency across the five test sessions of the data collection period. Estimated effects of test session make it possible to pinpoint those topics and also the precise time interval in which the change in the topic frequency took place. This analysis directly pursued one of the paper’s goals to characterize the psychological outcomes of the COVID-19 pandemic in older adults.

We fitted a structural topic model (STM) using the same data as in Analyses 2–4. The only difference between this model and the STM reported above is an inclusion of the story type × test session interaction as a meta-data covariate that may affect topical prevalence, i.e., the frequency with which a given topic is discussed. The model outcome is the prevalence of a topic estimated across story types and test sessions, along with inferential statistics.

This section focuses on the story types pertaining to autobiographical memories, namely “past”, “yesterday” and “future”. In each of the story types, a statistically significant temporal change (at the 5% level) was indicated for at least one topic, detailed below. When a significant change in topic prevalence did occur, its character was highly consistent. In most cases, test sessions t1–t2 were significantly different from sessions t3–t5, while no difference between sessions was found within either set of timepoints. This suggests that written texts contributed before the pandemic and in its first months (t1–t2) were highly similar in the selection and coverage of topics that participants chose. The shift in the topical prevalence typically took place between t2 and t3 (April 2020 and June 2020)—several months into the pandemic—and has remained relatively constant throughout the remainder of the data collection period. This temporal pattern converges perfectly with our analyses of affective and sensorimotor semantics in CoSoWELL narratives (Kyröläinen & Kuperman, 2020) and provides empirical material for testing recent theories on the long-term COVID-19 impact on autobiographical memories (Brown, 2021).

**Fig. 9:**
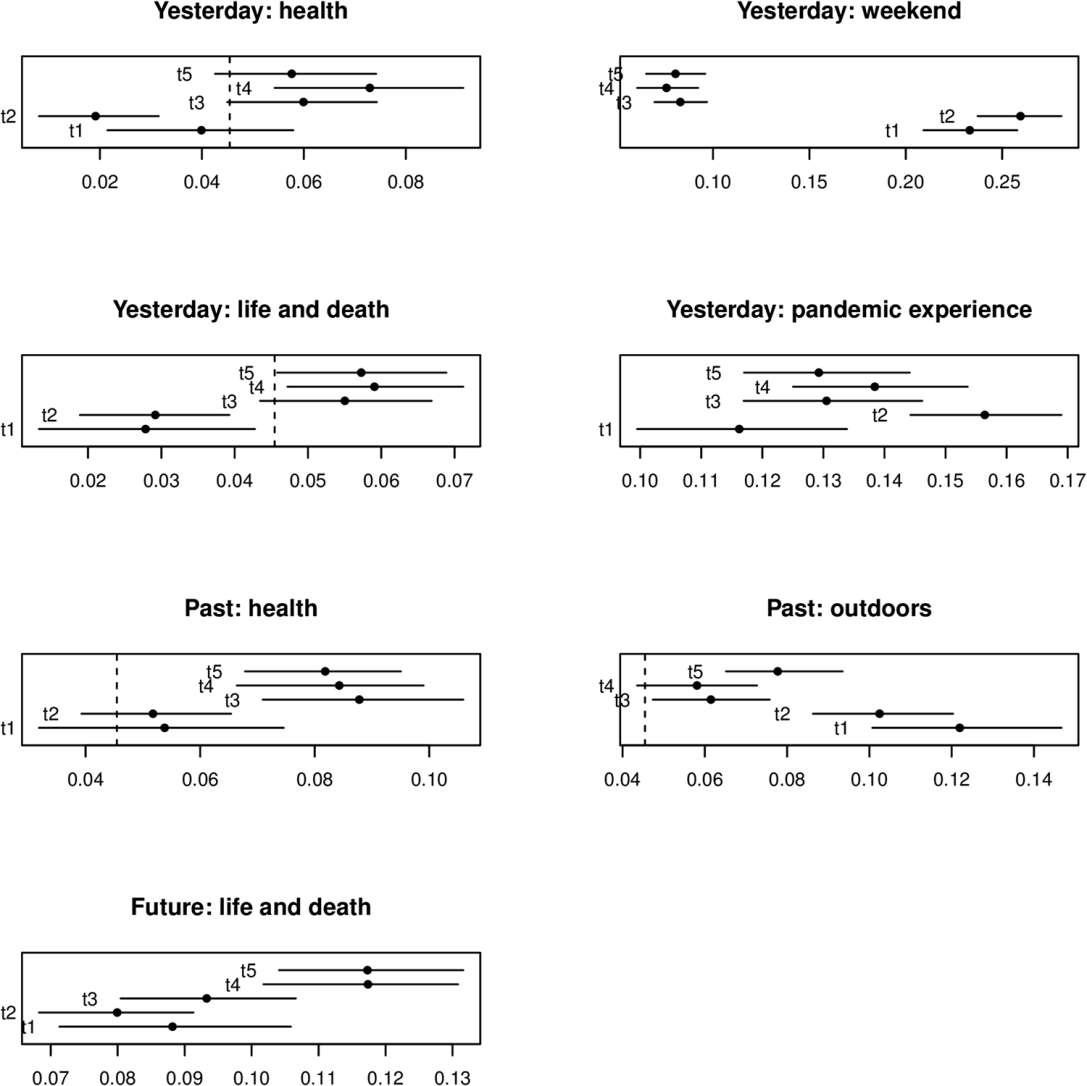
Topic prevalence by test session. Panel titles indicate Story type: topic name. *Error bars* stand for 95% CI. *Dotted lines* mark the baseline prevalence. The test sessions correspond to the following dates: t1 = 2019; t2 = from 2020-04-08 to 2020-06-16; t3 = from 2020-06-17 to 2020-06-30; t4 = from 2020-10-14 to 2020-11-05 and t5 = from 2021-01-12 to 2021-02-15

Figure 9 summarizes topics which demonstrated a significant (at the 5% level) change in prevalence over time. A useful datum in interpreting these results is the baseline prevalence of a topic, e.g., the frequency with which an average topic is discussed if all topics are equally distributed over the narratives. In our data, the baseline prevalence was 1/22 = 4.5%: Where it falls within the plot range, it is marked as a vertical dotted line in respective panels of Fig. 9.

Most observed changes took place in narratives about recent past, i.e., the story type “yesterday” (two top rows of Fig. 9). Topics labeled health and life and death saw a drastic increase in prevalence between sessions t2 and t3, while topic weekend saw an opposite change—to a much lower relative frequency—in the same time interval cf., (McKinnon et al., 2014). This reveals that the experience of the pandemic led to an increased focus on medical issues and threats to livelihood and a diminished role of entertainment and leisure in perception of the recent past among older adults. The one exception to the timeline of the change in content was associated with the topic labeled pandemic experience (second row right panel). The maximum prevalence was observed at t2, in the first few weeks of the global lockdown in North America, when pandemic experiences were most novel and salient in collective consciousness and discourse. For interpretability of topic labels in this section, we refer the reader to Fig. 3 which reports each topic as a word cloud of the 20 most diagnostic keywords.

A semantically similar shift characterized story type “past” in Fig. 9 (third row). Even though the narratives of this type related an event in a person’s distant past, which COVID-19 did not affect, the influence of the pandemic was visible in verbalization of distant past. Similar to story type “yesterday”, a significant increase in prevalence was observed in topic health, with a step-wise change between test sessions t2 and t3. Also, topic outdoors showed a drop in prevalence in the same time interval, supporting the finding above that issues of entertainment and leisure withdrew from the public discourse as the pandemic unfolded.

Finally, story type “future” (bottom row of Fig. 9)—which arguably reflects future outlook—incorporated a more frequent discussion of the topic we labeled life and death. While similar to the pattern found in story type “yesterday”, the increase in prevalence of this topic took place at a later time period, between test sessions t3–t4 (rather than t2–t3). This suggests a possibility that topics relevant for perception of recent events take time to affect the future outlook and the language that is used to describe it. If this hypothesis is correct, subsequent test sessions may reveal that story type “future” replicates changes in topical prevalence of story type “yesterday” but in a staggered way, with a delay.

In sum, Analysis 5 demonstrated the CoSoWELL corpus to be a valid source of data for studies interested in both the content of autobiographic memories and their change due to the psychological impact of the pandemic.

## General discussion

This paper presents the Cognitive and Social Well-Being (CoSoWELL) project which consists of two components. The one component in the focus of the present analyses is the corpus, a collection of English-written narratives elicited from North American older adults and supplemented by the second component, i.e., demographic and psychological participant data collected through the survey. The original goal of the project was to create a resource that would enable researchers to identify linguistic markers of age, retirement, social isolation, and loneliness in written productions both longitudinally within-participants and between participants. The main anticipated use of the resource was to examine how patterns of language use are affected by processes of aging and psychological states related to the intensity and nature of one’s verbalization of lived experiences and whether these patterns can be used predictively or diagnostically. The onset of the COVID-19 pandemic and the lockdown brought about the oft-reported exacerbation of feelings of loneliness and social isolation, especially in older adults who require a more thorough isolation and physical distancing because of greater mortality (Lebrasseur et al., 2021). The CoSoWELL project pivoted to incorporate an additional goal of characterizing the psychological outcomes of the COVID-19 pandemic, and its specific impact on one’s autobiographical memories and perception of the distant past, the recent past and future outlook (Brown, 2021). The present paper lays the groundwork for achieving these goals by introducing the first release of the project (CoSoWELL version 1.0) and validating the theoretical premises that went into design decisions for creating the CoSoWELL corpus (see below). We also offer an overview of future research directions that CoSoWELL data may aid in pursuing, beyond the scope of this methodological paper.

The current release of the corpus (CoSoWELL version 1.0) is large as it covers over 1.3 million tokens contributed by 1178 unique participants. A unique feature of the corpus is that it represents a time-series of five test sessions with repeated measures from roughly half of the participants and has a time coverage from before the COVID-19 pandemic through the first year of the global lockdown. Data collection is ongoing and additional corpus releases are planned (see below). At each test session, narratives were elicited in the same controlled way, using prompts that instructed participants to write a total of four narratives. Prompts for three of the story types—writing about a significant life event in the distant past, describing their day yesterday, and describing an anticipated event in the future—were chosen because they anchored narratives relative to veridical time, namely distant past, yesterday, and future, and were expected to tap into different facets of the writers’ autobiographical memories. Only certain memories are significant and can become part of an individual’s life story.

We anticipated and then observed that story type “yesterday” was the most revealing regarding the psychological impact of the pandemic. Narratives related to future—and the formal and semantic choices at the lexical and topical level made in writing them—provide yet another complementary window into the nexus of narratives and language use, pointing to the writer’s future outlook. Additionally, the corpus contains stories written as a description of the Cookie Theft picture that is widely used in clinical studies as a standard tool for diagnosing cognitive well-being (for an overview see (Cummings, 2019) and also the Introduction for discussion). The function of the story type “cookie” was to serve as a control/baseline condition, against which to compare narratives produced under the other three story types, both within a given test session and over time.

The utility of the CoSoWELL project to achieve the proposed goals hinged on the validity of its narratives as a linguistic window into psychological states related to diverse facets of autobiographical memories and to the ongoing pandemic. This paper set out to explore this validity in a series of analyses of the linguistic structure and the content of the narratives in the corpus. First, using regression modeling and structural topic modeling (STM), we showed that the prompts associated with the four chosen story types elicited narratives that were distinct and separable in their lexico-syntactic properties (Analysis 1) and the nature and composition of their topics (Analysis 2). As the next step, Analysis 3 used a random forests machine learning technique to prove that the topics estimated by the STM provided a very accurate (89%) classification of individual narratives into story types. Importantly, the STM that learned the topic structure from the narratives only had exposure to word counts and was blind to all the meta-data regarding those narratives, including what story type they were elicited under. This strongly suggests both a formal and functional separability of the four story types.

This separability is useful only to the extent that each story type taps into a relevant facet of autobiographical memory. Analysis 4 presented the variable importance of the predictors based on the random forests. This highlighted the topics that were most influential for the accurate classification of narratives into story types. For each story type related to autobiographical memory (“past”, “yesterday”, and “future”), these topics showed an excellent convergence with the prior literature on the content of autobiographical memories or—in the case of rare catastrophic events like the pandemic—with the researchers’ subjective intuitions about the topics that are at the forefront of the writer’s mind. Writing about distant past thus evoked topics like family life and family members, as well as education, all described in prior memory research as central anchoring themes of one’s self-perception (e.g., Thomsen et al., 2011).

Future outlook highlighted such topics as adventure and family events, as well as retirement with its obvious relevance to many participants in our age group of 55+ (e.g., Thomsen & Berntsen, 2008; Thomsen et al., 2011). Topics central for stories about recently lived experiences (“yesterday”) were directly tied to the COVID-19 pandemic, which is not surprising given the salience of this ongoing event. On the basis of computational analyses, we conclude that the content of the CoSoWELL narratives is reflective both of entrenched autobiographical memories and also of the dynamic circumstances of the writers’ day-to-day life. Thus, the corpus is a valid source of psychologically relevant data for pursuing the first goal of the project, i.e., studying the effect of age and social context on the formal and semantic structure of language use in English-speaking older adults.

We also contributed to the second goal of the project by characterizing some aspects of the psychological impact of the COVID-19 pandemic. Analysis 2 uncovered two topics (pandemic life and pandemic experience) directly related to lexical items that were either specifically coined or rose in their frequency of use since the start of the pandemic. Analysis 4 further indicated that these topics were the ones that discriminated the most between the writer’s experience of recent events (story type “yesterday”) from other experiences. The clearest insight into the impact of the pandemic was borne out in Analysis 5, where a structural topic model was fitted to individual narratives with a story type × test session interaction as a covariate. This analysis determined changes in topic prevalence—how often the topic is discussed—over time for each story type. The main findings were a drastic increase in the prevalence of topics related to health and well-being and a parallel decrease in the topic prevalence related to leisure and entertainment. Narratives about the recent past (“yesterday”) also indicated a direct impact of the pandemic experience by showing a prevalence increase in respective topics. Yet, these changes were observed in all autobiographical story types. Even though the COVID-19 pandemic did not affect the writers’ past and may not constitute a sizable part of their future, it has clearly influenced their perception of self and led to a sweeping revisiting of one’s personal, lived experiences.

Beyond outlining the nature of the changes in narratives (and by implication, psychological states) over time, Analysis 5 generated a time-locked account of when exactly these changes took place. With only few exceptions, a shift in topic prevalence—arguably mirroring a shift in the public consciousness—occurred some 4 months after the onset of the global lockdown in North America, in the interval between test session t2 (April 2020) and t3 (June 2020). The one exception was the topic we labeled pandemic experience, which increased in prevalence already at t2, e.g., 3 weeks after the lockdown took place. The data also suggest that the same topics that characterize one’s perception of recent events migrate to one’s future outlook, but with a possible delay in time. Taken together, these findings quantify the degree of inertia in the psychological response to the COVID-19 pandemic and can be used comparatively by researchers interested in the public response and recollection of other catastrophic events, e.g., the 9/11 terrorist attack, school shootings, or prior pandemics (Barber & Mather, 2014; Fischer-Preßler, Schwemmer, & Fischbach, 2019; Cohn et al., 2004; Luhmann & Bleidorn, 2018; McKinnon et al., 2014). Thus, the CoSoWELL corpus is a rich and novel source of data regarding the psychological state of older adults and its change over the course of the pandemic.

## Limitations and future directions

We view the key role of the present paper as an introduction to the CoSoWELL project and an empirical validation of the design decisions and theoretical premises. By opening this resource to the research community, we aim at boosting research into the cognitive and social well-being of older adults, with an emphasis on the psychological impact of the COVID-19 pandemic.

As stated in the Introduction, CoSoWELL data offer more research possibilities than this paper covers. A critical question, which can be answered with the presently published demographic data in hand, is the link between story content (including its change throughout the COVID-19 pandemic) and such characteristics as age, gender, education, and place of residence of the writer. The influence of demographic parameters on attitudes towards the pandemic and severity and nature of its psychological impact on mental health is at the forefront of current research (e.g., Bernabe-Valero, Melero-Fuentes, De Lima Argimon, & Gerbino, 2021; Carstensen, Shavit, & Barnes, 2020; Kyröläinen, Luke, Libben, & Kuperman, 2021; McElroy et al., 2020). Moreover, with Canada and the USA as two (unequal) sources of data, it may be feasible to link the dynamics of narrative change in writers from these countries to the different unfolding of the pandemic in those countries (Jaggers, Gillet, Kuperman, Kyröläinen, & Sonnadara, 2022; Pickup, Stecula, & Van Der Linden, 2020). We anticipate that examination of the affective and sensorimotor dimensions of the narratives produced before and during the pandemic (Kyröläinen & Kuperman, 2020) against extra-linguistic data on the writers will enable researchers to formalize and quantify this link.

Future work will be further facilitated by the planned releases of the CoSoWELL corpus, which will include data from psychological surveys from participants, including their self-rated estimates of loneliness, social isolation and memory functioning. Together with the demographic data, CoSoWELL materials will reveal how the strength of one’s social network and social engagement modulate one’s experience of the pandemic, as revealed in patterns of language use.

While our own methodological toolkit leans towards quantitative analyses of linguistic data, CoSoWELL stories readily lend themselves to qualitative analysis. For instance, these stories can reveal sources of emotional and cognitive resilience that older adults showed under the pandemic (Jaggers et al., 2022). They can also point to resources and experiences that helped older adults endure and overcome the major disruption of their lives. The stories may also reveal how critical the ability to stay connected was for coping with the impact of the pandemic. Plausibly, the demographic section of CoSoWELL will make it possible to analyze differences between types of communities (e.g., urban vs. rural) or even communities in a specific locale. Thus, we foresee a fruitful use of content analysis linking stories to personal and community characteristics of the writers^1^.

The CoSoWELL project is not without limitations. A central one is that it is based on a self-selected sample of North American older adults (55+) who have technology, knowledge, and means to access one of the crowdsourcing platforms (Amazon’s Mechanical Turk or Prolific) to participate in data collection. Thus, the resource is built on data that do not cross the “digital divide” and thus is not representative of those older adults who have suffered the most from consequences of digital inequity and its implications for social and physical isolation and mental health (Figueroa & Aguilera, 2020; Van Jaarsveld, 2020). Another limitation—driven by the imbalance in national representation in crowdsourcing platforms—is a skew towards US-based rather than Canadian-based participants, making difficult a comparative analysis of these two countries.

In conclusion, a detailed understanding of the factors that contribute to or, conversely, harm cognitive and social well-being of older adults is a goal of academic, social, and economic importance given the growing percentage of the aging population in North America and world-wide. This importance has increased multi-fold due to the medical and psychological impact of the COVID-19 pandemic. The physical and social isolation indicated as counter-measures to the pandemic greatly exacerbated feelings of loneliness and anxiety in this age group. We propose that the CoSoWELL corpus is a valuable and valid resource to address some of the most present and pressing challenges that psychological science faces today.

## Availability

The CoSoWELL project (version 1.0) is publicly available and can be downloaded from the Open Science Framework at https://osf.io/x4s28/?view_only=659fc5216ca1477e801306d05ac0feaa (the current version is for reviewing purposes only; full access will be given after acceptance). The website https://akkyro.gitlab.io/project/cosowell/ will provide all updates related to new data releases, data collection and publications. As part of this release, the following files are made publicly available.

- A compressed corpus stored as an R data frame (cosowell_corpus_v1.Rds
- A compressed corpus stored as a tab-delimited text file (cosowell_corpus_v1.txt.gz)
- A compressed “matched” corpus (with data from participants who completed both the narrative writing and the survey tasks) stored as an R data frame (cosowell_corpus_matched_v1.Rds)
- A compressed “matched” corpus (with data from participants who completed both the narrative writing and the survey tasks) stored as a tab-delimited text file (cosowell_corpus_matched_v1.txt.gz)
- A compressed survey stored as an R data frame (cosowell_survey_v1.Rds)
- A compressed survey stored as a tab-delimited text file (cosowell_survey_v1.txt.gz)
- Document-topic distribution as an R file (doc_topic_dist_v1.Rds) and as a tab-delimited text file (doc_topic_dist_v1.tsv)
- Topical keywords as an R file (topical_keywords_v1.Rds) and as a tab-delimited text-file (topical_keywords_v1.tsv)

Any future release will have its own version number and different URL to ensure the ease of availability of the data and the replicability of studies relying on a specific version of the corpus.

## Supplementary Information

ESM 1 (PDF 126 KB)

ESM 2 (PDF 236 KB)

## Footnotes

1 We thank the anonymous reviewer for raising this point.

## References

[CR1] Agis D, Goggins MB, Oishi K, Oishi K, Davis C, Wright A, Kim EH, Sebastian R, Tippett DC, Faria A, Hillis AE (2016). Picturing the size and site of stroke with an expanded National Institutes of Health Stroke Scale. Stroke.

[CR2] Airoldi EM, Bischof JM (2016). A regularization scheme on word occurrence rates that improves estimation and interpretation of topical content. Journal of the American Statistical Association.

[CR3] Alea N, Bluck S (2003). Why are you telling me that? A conceptual model of the social function of autobiographical memory. Memory.

[CR4] Arnhold A, Kyröläinen A-J (2017). Modelling the interplay of multiple cues in prosodic focus marking. Laboratory Phonology: Journal of the Association for Laboratory Phonology.

[CR5] Ash S, Ternes K, Bisbing T, Min NE, Moran E, York C, McMillan CT, Irwin DJ, Grossman M (2016). Dissociation of quantifiers and object nouns in speech in focal neurodegenerative disease. Neuropsychologia.

[CR6] Atance CM, O’Neill DK (2005). The emergence of episodic future thinking in humans. Learning and Motivation.

[CR7] Baayen, R. H. (2001). Word frequency distributions. Dordrecht: Kluwer Academic Publishers.

[CR8] Barber SJ, Mather M (2014). How retellings shape younger and older adults’ memories. Journal of Cognitive Psychology.

[CR9] Bartlett FC (1932). Remembering: A study in social and experimental psychology.

[CR10] Benoit K, Watanabe K, Wang H, Nulty P, Obeng A, Müller S, Matsuo A (2018). quanteda: An R package for the quantitative analysis of textual data. Journal of Open Source Software.

[CR11] Berna F, Potheegadoo J, Aouadi I, Ricarte JJ, Alle MC, Coutelle R, Boyer L, Cuervo-Lombard CV, Danion J-M (2016). A meta-analysis of autobiographical memory studies in schizophrenia spectrum disorder. Schizophrenia Bulletin.

[CR12] Bernabe-Valero G, Melero-Fuentes D, De Lima Argimon II, Gerbino M (2021). Individual differences facing the COVID-19 pandemic: the role of age, gender, personality, and positive psychology. Frontiers in Psychology.

[CR13] Biecek P (2018). DALEX: Explainers for Complex predictive models in R. Journal of Machine Learning Research.

[CR14] Bischof, J. M., & Airoldi, E. M (2012). Summarizing topical content with word frequency and exclusivity.

[CR15] Bjursell C (2019). Growth through education: The narratives of older adults. Frontiers in Sociology.

[CR16] Blei DM, Ng AY, Jordan MI (2003). Latent Dirichlet allocation. Journal of machine Learning research.

[CR17] Bohn A, Berntsen D (2011). The reminiscence bump reconsidered: Children’s prospective life stories show a bump in young adulthood. Psychological Science.

[CR18] Boyd, R. L., Wilson, S. R., Pennebaker, J. W., Kosinski, M., Stillwell, D. J., & Mihalcea, R. (2015). Values in words: Using language to evaluate and understand personal values. In *Proceedings of the Ninth International AAAI Conference on Web and Social Media. (pp. 31–40)*.

[CR19] Breiman L (2001). Random forests. Machine Learning.

[CR20] Brooks SK, Webster RK, Smith LE, Woodland L, Wessely S, Greenberg N, Rubin GJ (2020). The psychological impact of quarantine and how to reduce it: Rapid review of the evidence. The Lancet.

[CR21] Brown NR (1990). Organization of public events in long-term memory.. Journal of Experimental Psychology: General.

[CR22] Brown NR (2021). The possible effects of the COVID-19 pandemic on the contents and organization of autobiographical memory: a transition-theory perspective. Cognition.

[CR23] Brown NR, Lee PJ (2010). Public events and the organization of autobiographical memory: an overview of the living-in-history project. Behavioral Sciences of Terrorism and Political Aggression.

[CR24] Brown NR, Schweickart O, Svob C (2016). The effect of collective transitions on the organization and contents of autobiographical memory: a transition theory perspective. American Journal of Psychology.

[CR25] Brown, N. R., Shevell, S. K., & Rips, L. J. (1986). Public memories and their personal context. In DC Rubin (Ed.) *Autobiographical memory (pp. 137–158). Cambridge University Press*.

[CR26] Carstensen LL, Shavit YZ, Barnes JT (2020). Age advantages in emotional experience persist even under threat from the COVID-19 pandemic. Psychological Science.

[CR27] Chafe, W. L. (Ed.) (1980). *The Pear Stories: Cognitive, cultural, and linguistics aspects of narrative production*. Norwood: ABLEX Publishing Corporation.

[CR28] Chafe, W. L. (1994). Discourse, consciousness, and time. The flow and displacement of conscious experience in speaking and writing, University of Chicago Press, Chicago.

[CR29] Cohn MA, Mehl MR, Pennebaker JW (2004). Linguistic markers of psychological change surrounding September 11, 2001. Psychological Science.

[CR30] Conway MA (2005). Memory and the self. Journal of Memory and Language.

[CR31] Croft, W. (2017). Using typology to develop guidelines for Universal Dependencies. Gothenburg, Sweden.

[CR32] Croft, W., Nordquist, D., Looney, K., & Regan, M. (2017). Linguistic typology meets Universal Dependencies. In M. Dickinson, J. Hajic, S. Kübler, & A. Przepiórkowski (Eds.) *15th International Workshop on Treebanks and Linguistic Theories* (p. 6375). Bloomington: IN: CEUR Workshop Proceedings.

[CR33] Cummings L (2019). Describing the Cookie Theft picture: Sources of breakdown in Alzheimer’s dementia. Pragmatics and Society.

[CR34] de Marneffe M-C, Nivre J (2019). Dependency grammar. Annual Review of Linguistics.

[CR35] Donald M (2001). A mind so rare: The evolution of human consciousness.

[CR36] Eichstaedt JC, Smith RJ, Merchant RM, Ungar LH, Crutchley P, Preotiuc-Pietro D, Asch DA, Schwartz HA (2018). Facebook language predicts depression in medical records. Proceedings of the National Academy of Sciences.

[CR37] Eyigoz E, Mathur S, Santamaria M, Cecchi G, Naylor M (2020). Linguistic markers predict onset of Alzheimer’s disease. EClinicalMedicine.

[CR38] Figueroa CA, Aguilera A (2020). The need for a mental health technology revolution in the COVID-19 pandemic. Frontiers in Psychiatry.

[CR39] Fischer-Preßler D, Schwemmer C, Fischbach K (2019). Collective sense-making in times of crisis: connecting terror management theory with twitter reactions to the Berlin terrorist attack. Computers in Human Behavior.

[CR40] Fivush R (2008). Remembering and reminiscing: how individual lives are constructed in family narratives. Memory Studies.

[CR41] Fivush R (2011). The development of autobiographical memory. Annual Review of Psychology.

[CR42] Friedman, J. H. (2001). Greedy function approximation: A gradient boosting machine. Annals of Statistics, 11891232.10.1214/aos/1013203451

[CR43] Goodglass, H., Kaplan, E., & Barresi, B. (2001). Boston diagnostic aphasia examination-third edition (BDAE-3). Philadelphia: PA (Lippincott Williams & Wilkins.

[CR44] Graesser AC, McNamara DS, Kulikowich JM (2011). Coh-Metrix: Providing multilevel analyses of text characteristics. Educational Researcher.

[CR45] Hall, D., Jurafsky, D., & Manning, C. D (2008). Studying the history of ideas using topic models. In *Proceedings of the 2008 conference on empirical methods in natural language processing, (p. 363–371)*.

[CR46] Hand DJ, Till RJ (2001). A simple generalisation of the area under the ROC curve for multiple class classification problems. Machine Learning.

[CR47] Ho FK, Petermann-Rocha F, Gray SR, Jani BD, Katikireddi SV, Niedzwiedz CL, Foster H, Hastie CE, Mackay DF, Gill JMR (2020). Is older age associated with COVID-19 mortality in the absence of other risk factors? General population cohort study of 470,034 participants. PloS one.

[CR48] Holmes, E. A., O’Connor, R. C., Perry, V. H., Tracey, I., Wessely, S., Arseneault, L., ..., et al. (2020). Multidisciplinary research priorities for the COVID-19 pandemic: a call for action for mental health science. *The Lancet Psychiatry*. 10.1016/S2215-0366(20)30168-1 10.1016/S2215-0366(20)30168-1 PMC7159850 32304649

[CR49] Hughes ME, Waite LJ, Hawkley LC, Cacioppo JT (2004). A short scale for measuring loneliness in large surveys. Research on Aging.

[CR50] Jagaiah T, Olinghouse NG, Kearns DM (2020). Syntactic complexity measures: Variation by genre, grade-level, students’ writing abilities, and writing quality. Reading and Writing.

[CR51] Jaggers, K., Gillet, J., Kuperman, V., Kyröläinen, A.-J., & Sonnadara, R. (2022). Personhood and aging: Exploring the written narratives of older adults as articulations of personhood in later life. Journal of Aging Studies (submitted). 10.1016/j.jaging.2022.101040 36008023

[CR52] Kenyon GM, Gar M, Randall WL (1999). Introduction: Narrative gerontology. Journal of Aging Studies.

[CR53] Kuperman, V., Jarema, G., & Libben, G. (2021). The mental lexicon as polylogue. In G. Libben, G. Jarema, & V. Kuperman (Eds.) *Polylogues on The Mental Lexicon: An exploration of fundamental issues and directions* (pp. 1–16): John Benjamins.

[CR54] Kyle, K. (2019). Measuring lexical richness. In S. Webb (Ed.) *The Routledge handbook of vocabulary studies* (p. 454475): Routledge.

[CR55] Kyröläinen, A. -J., & Kuperman, V. (2020). Temporal dynamics of affect and loneliness in older adults in the first year of the COVID-19 pandemic: Insights from the Cognitive and Social Well-Being (CoSoWell) corpus. 10.1080/0361073X.2023.2219188 37270799

[CR56] Kyröläinen, A.-J., Luke, J., Libben, G., & Kuperman, V. (2021). Valence norms for 3,600 English words collected during the COVID-19 pandemic: Effects of age and the pandemic. Behavior Research Methods, 1–12. 10.3758/s13428-021-01740-0 PMC8676940 34918233

[CR57] Labov W (1972). Language in the inner city.

[CR58] Labov, W., & Waletzky, J. (1967). Narrative analysis: Oral versions of personal experience. In J. Helm (Ed.) *Essays on the verbal and visual arts* (p. 1244). Seattle, Washington: University of Washington Press.

[CR59] Lebrasseur A, Fortin-Bédard N, Lettre J, Raymond E, Bussières E-L, Lapierre N, Faieta J, Vincent C, Duchesne L, Ouellet M-C, Gagnon E, Tourigny A, Lamontagne M-È, Routhier F (2021). Impact of the COVID-19 pandemic on older adults: rapid review. JMIR Aging.

[CR60] Lempert KM, MacNear KA, Wolk DA, Kable JW (2020). Links between autobiographical memory richness and temporal discounting in older adults. Scientific Reports.

[CR61] Linde C (1993). Life stories: The creation of coherence.

[CR62] Liu L, Tang L, Dong W, Yao S, Zhou W (2016). An overview of topic modeling and its current applications in bioinformatics. SpringerPlus.

[CR63] Luhmann M, Bleidorn W (2018). Changes in affect, cognition, and perceived behavioral changes among vicarious victims of the Paris terrorist attacks of November 13, 2015. Social Psychological and Personality Science.

[CR64] Mairesse François, Walker MA, Mehl MR, Moore RK (2007). Using linguistic cues for the automatic recognition of personality in conversation and text. Journal of Artificial Intelligence Research.

[CR65] Matsuki K, Kuperman V, Van Dyke JA (2016). The random forests statistical technique: An examination of its value for the study of reading. Scientific Studies of Reading.

[CR66] McAdams DP, Bauer JJ, Sakaeda AR, Anyidoho NA, Machado MA, Magrino-Failla K, White KW, Pals JL (2006). Continuity and change in the life story: A longitudinal study of autobiographical memories in emerging adulthood. Journal of Personality.

[CR67] McElroy E, Patalay P, Moltrecht B, Shevlin M, Shum A, Creswell C, Waite P (2020). Demographic and health factors associated with pandemic anxiety in the context of COVID-19. British Journal of Health Psychology.

[CR68] McKinnon MC, Palombo DJ, Nazarov A, Kumar N, Khuu W, Levine B (2014). Threat of death and autobiographical memory. Clinical Psychological Science.

[CR69] Mimno, D., Wallach, H., Talley, E., Leenders, M., & McCallum, A. (2011). Optimizing semantic coherence in topic models. In *Proceedings of the 2011 conference on empirical methods in natural language processing (pp. 262–272)*.

[CR70] Nelson K (1996). Language in cognitive development: the emergence of the mediated mind (Vol. 1).

[CR71] Nelson K, Fivush R (2004). The emergence of autobiographical memory: A social cultural developmental theory. Psychological Review.

[CR72] Nippold MA, Cramond PM, Hayward-Mayhew C (2014). Spoken language production in adults: Examining age-related differences in syntactic complexity. Clinical Linguistics & Phonetics.

[CR73] Nivre, J. (2015). Towards a universal grammar for natural language processing. In Alexander Gelbukh (Ed.) *Computational linguistics and intelligent text processing* (p. 316). New York: Springer.

[CR74] Nucci M, Mapelli D, Mondini S (2012). Cognitive Reserve Index questionnaire (CRIq): A new instrument for measuring cognitive reserve. Aging Clinical and Experimental Research.

[CR75] Pennebaker JW, King LA (1999). Linguistic styles: Language use as an individual difference. Journal of Personality and Social Psychology.

[CR76] Pickup M, Stecula D, Van Der Linden C (2020). Novel coronavirus, old partisanship: COVID-19 attitudes and behaviours in the United States and Canada. Canadian Journal of Political Science/Revue canadienne de science politique.

[CR77] Prebble SC, Addis DR, Tippett LJ (2013). Autobiographical memory and sense of self. Psychological Bulletin.

[CR78] Priva UC, Austerweil JL (2015). Analyzing the history of cognition using topic models. Cognition.

[CR79] R Core Team (2020). R: A language and environment for statistical computing. Vienna.

[CR80] Raina, P., Wolfson, C., Kirkland, S., Griffith, L. E., Balion, C., Cossette, B, ..., Young, L. (2019). Cohort profile: The Canadian Longitudinal Study on Aging (CLSA). International Journal of Epidemiology. 10.1093/ije/dyz173 10.1093/ije/dyz173 PMC6929533 31633757

[CR81] Randall WL, Kenyon GM, Gary M (2004). Time, story, and wisdom: Emerging themes in narrative gerontology. Canadian Journal on Aging/La Revue canadienne du vieillissement.

[CR82] Rathbone CJ, Holmes EA, Murphy SE, Ellis JA (2015). Autobiographical memory and well-being in aging: the central role of semantic self-images. Consciousness and Cognition.

[CR83] Reese E, Haden CA, Baker-Ward L, Bauer P, Fivush R, Ornstein PA (2011). Coherence of personal narratives across the lifespan: A multidimensional model and coding method. Journal of Cognition and Development.

[CR84] Renoult L, Rugg MD (2020). An historical perspective on Endel Tulving’s episodic-semantic distinction. Neuropsychologia.

[CR85] Roberts ME, Stewart BM, Airoldi EM (2016). A model of text for experimentation in the social sciences. Journal of the American Statistical Association.

[CR86] Roberts ME, Stewart BM, Tingley D (2019). stm: An R package for structural topic models. Journal of Statistical Software.

[CR87] Roberts, M. E., Stewart, B. M., Tingley, D., & Airoldi, E. M. (2013). The structural topic model and applied social science. In *Advances in neural information processing systems workshop on topic models: Computation, application, and evaluation*.

[CR88] Roberts ME, Stewart BM, Tingley D, Lucas C, Leder-Luis J, Gadarian SK, Shana K, Albertson B, Rand DG (2014). Structural topic models for open-ended survey responses. American Journal of Political Science.

[CR89] Schwartz HA, Eichstaedt JC, Kern ML, Dziurzynski L, Ramones SM, Agrawal M, Shah A, Kosinski M, Stillwell D, Seligman MEP, Ungar LH (2013). Personality, gender, and age in the language of social media: The open-vocabulary approach. PLoS ONE.

[CR90] Semin GR, Smith ER (1999). Revisiting the past and back to the future: Memory systems and the linguistic representation of social events. Journal of Personality and Social Psychology.

[CR91] Shahid Z, Kalayanamitra R, McClafferty B, Kepko D, Ramgobin D, Patel R, Aggarwal CS, Vunnam R, Sahu N, Bhatt D (2020). COVID-19 and older adults: what we know. Journal of the American Geriatrics Society.

[CR92] Shum MS (1998). The role of temporal landmarks in autobiographical memory processes. Psychological Bulletin.

[CR93] Smith G, Sala SD, Logie RH, Maylor EA (2000). Prospective and retrospective memory in normal ageing and dementia: A questionnaire study. Memory.

[CR94] Stirman SW, Pennebaker JW (2001). Word use in the poetry of suicidal and nonsuicidal poets. Psychosomatic Medicine.

[CR95] Straka, M., & Straková, J. (2017). Tokenizing, POS tagging, lemmatizing and parsing UD 2.0 with udpipe. In *Proceedings of the CoNLL 2017 Shared Task: Multilingual Parsing from Raw Text to Universal Dependencies.* (p. 8899). 10.18653/v1/K17-3009

[CR96] Strobl C, Malley J, Tutz G (2009). An introduction to recursive partitioning: Rationale, application and characteristics of classification and regression trees, bagging and random forests. Psychological Methods.

[CR97] Sun, J., Schwartz, H. A., Son, Y., Kern, M. L. , & Vazire, S. (2020). The language of well-being: Tracking fluctuations in emotion experience through everyday speech. Journal of Personality and Social Psychology, 364–387. 10.1037/pspp0000244 10.1037/pspp0000244 30945904

[CR98] Tagliamonte SA, Baayen RH (2012). Model, forests and trees of York English: Was/were variation as a case study for statistical practice. Language Variation and Change.

[CR99] Thomsen DK, Berntsen D (2008). The cultural life script and life story chapters contribute to the reminiscence bump. Memory.

[CR100] Thomsen DK, Pillemer DB, Ivcevic Z (2011). Life story chapters, specific memories and the reminiscence bump. Memory.

[CR101] Trope Y, Liberman N (2003). Temporal construal. Psychological Review.

[CR102] Tulving, E. (1972). Episodic and semantic memory. In E. Tulving, & W. Donaldson (Eds.) *Organization of Memory* (pp. 381–403). New York: Academic Press.

[CR103] Tulving, E. (1983). Elements of episodic memory. Clarendon Press.

[CR104] Tulving E (1985). Memory and consciousness. Canadian Psychology/Psychologie canadienne.

[CR105] Van Jaarsveld, G. M. (2020). The effects of COVID-19 among the elderly population: a case for closing the digital divide. Frontiers in Psychiatry, 11. 10.3389/fpsyt.2020.577427 PMC7693633 33304283

[CR106] Vanaken, L., Bijttebier, P., Fivush, R., & Hermans, D. (2021). Narrative coherence predicts emotional well-being during the COVID-19 pandemic: a two-year longitudinal study. Cognition and Emotion, 1–12. 10.1080/02699931.2021.1902283 10.1080/02699931.2021.1902283 33734018

[CR107] Vermeer A (2000). Coming to grips with lexical richness in spontaneous speech data. Language Testing.

[CR108] Wickham H (2014). Tidy data. Journal of statistical software.

[CR109] Wright MN, Ziegler A (2017). ranger: A fast implementation of random forests for high dimensional data in C++ and R. Journal of Statistical Software.

[CR110] Yarkoni T (2010). Personality in 100,000 words: A large-scale analysis of personality and word use among bloggers. Journal of Research in Personality.

