# Supplementary material for: Cognitive and social well-being in older adulthood: The CoSoWELL corpus of written life stories
Source: Behav Res Methods. 2022 Aug 24;55(6):2885–909. doi: 10.3758/s13428-022-01926-0 (PMC9400578; doi:10.3758/s13428-022-01926-0)
Supplement: Supplementary file 1 — (PDF 126 KB) [file 13428_2022_1926_MOESM1_ESM.pdf]

Supplementary material: CoSoWELL survey and corpus

Aki-Juhani Kyröläinen
McMaster University and Brock University

James Gillett
McMaster University

Megan Karabin
McMaster University

Ranil Sonnadara
McMaster University and Vector Institute

Victor Kuperman
McMaster University

Supplementary material: CoSoWELL survey and corpus

Introduction

In this supplementary material, we provide the description of the variables available in the CoSoWELL project (release version 1.0). In the CoSoWELL survey section, we provide the label associated with each of the variables and, when appropriate, the question used to elicit the response as well as descriptive statistics of responses. Cases when derived variables are provided instead of the original responses are explicitly mentioned in the description of the variable. All the variables and the names associated with them are given in lower case. Missing values are encoded as NA. In the CosoWELL corpus section, we provide the label associated with each of the variables. Most of the variables provided in this section are related to the output of the output of the parser. In addition to providing a description of the variables, when appropriate, descriptive statistics are also reported. Each variable is described in a separate subsection.

CoSoWELL survey

Variable: id

Description: a unique identifier of the participants (N = 1,451). This variable can be used to link the survey data of a given participant with their written narrative, when available (n = 1028).

Variable: age

Description: A derived variable based on the date of birth and submission date. Only the derived variable age (in years) is provided with the release of the data. As part of the data collection, only the year and month were collected in order to comply with the requirement of the Research Ethics Board. In the derivation, the missing information pertaining to the day of the month was imputed with one.

Question: When were you born? Please use numbers (mm and yyyy) in the fields below.

Table 1

Summary information of the variable age, in years.

Min	Max	*M*	*SD*	*N*	NA
55.00	84.00	63.14	5.34	1451	0

Variable: phase

Description: A variable derived from the submission date indicating whether the data collection took place before or after the global COVID-19 lockdown (13-March-2020).

Table 2

Summary information of the variable phase.

	n
cov	996
pre_cov	455
NA	0

Variable: gender

Description: Participant gender.

Question: What is your gender?

Table 3

Summary information of the variable gender.

	n
female	946
male	500
other	3
prefer_not_to_say	2
NA	0

Variable: retired

Description: Participant's retirement status.

Question: Are you currently retired?

Table 4

Summary information of the variable retired.

	n
no	811
yes	640
NA	0

Variable: education

Description: The highest level of education.

Question: Please select the highest level of education you have completed.

Table 5

Summary information of the variable education.

	n
some high school	4
high school graduate	130
some college, no degree	323
associate's degree	207
bachelor's degree	488
master's degree	239
doctorate	47
NA	13

Variable: education2

Description: A derived variable of education where the infrequent levels have been combined.

Table 6

Summary information of the variable education2

	n
high_school	134
college	530
bachelor	488
graduate	286
NA	13

Variable: age_education

Description: The age of a given participant when the highest level of education was attained.

Question: How old were you when you finished your highest level of education?

Table 7

Summary information of the variable age_education.

Min	Max	*M*	*SD*	*N*	NA
0.00	68.00	28.05	10.84	1451	14

Variable: years_education

Description: Education of a given participant assessed in number of years.

Question: How many years of education do you have in total so far (include all schooling and vocational or professional training)?

Table 8

Summary information of the variable years_education.

Min	Max	*M*	*SD*	*N*	NA
0.00	60.00	16.47	4.28	1451	3

Variable: read_ability

Description: Self-assessed reading ability on a five-point scale.

Question: How would you rate your everyday reading ability?

Table 9

Summary information of the variable read_ability

	n
poor	8
fair	39
good	204
very_good	472
excellent	723
NA	5

Variable: write_ability

Description: Self-assessed writing ability on a five-point scale.

Question: How would you rate your everyday writing ability?

Table 10

Summary information of the variable write_ability.

	n
poor	8
fair	56
good	280
very_good	545
excellent	552
NA	10

Variable: speak_habit

Description: Self-assessed communication frequency.

Question: How many hours a day do you communicate with people, in person, on the phone or via the Internet?

Table 11

Summary information of the variable speak_habit.

Min	Max	*M*	*SD*	NOBS	NA
0.00	10.00	2.63	2.25	1451	51

Variable: read_print_habit

Description: Self-reported frequency of reading printed materials.

Question: How many hours a day do you read books, magazines or newspapers?

Table 12

Summary information of the variable read_print_habit.

Min	Max	*M*	*SD*	NOBS	NA
0.00	12.00	1.77	1.34	1451	5

Variable: read_web_habit

Description: Self-assessed reading habit of materials on the Internet.

Question: How many hours a day do you read materials on the Internet?

Table 13

Summary information of the variable read_web_habit.

Min	Max	*M*	*SD*	NOBS	NA
0.00	11.00	2.47	1.79	1451	21

Variable: num_friends

Description: Number of friends (self-reported).

Question: How many friends would you say you have?

Table 14

Summary information of the variable num_friends.

Min	Max	*M*	*SD*	NOBS	NA
0.00	800.00	15.32	40.78	1451	3

Variable: social_meet_freq

Description: Recent frequency of social meetings on a seven-point scale.

Question: In the past 12 months, how often did you get together socially with friends or relatives?

Table 15

Summary information of the variable social_meet_freq.

	n
never	62
less_than_once_a_year	56
about_once_or_twice_a_year	138
several_times_a_year	234
about_once_a_month	388
every_week	411
several_times_a_week	159
NA	3

Variable: professional_meet_freq

Description: Recent frequency of social meetings in a professional setting on a seven-point scale.

Question: In the past 12 months, how often did you attend meetings of any organized group? (Examples include, a choir, a committee or board, a support group, a sports or exercise group, a hobby group, or a professional society.)

Table 16

Summary information of the variable professional_meet_freq.

	n
never	487
less_than_once_a_year	110
about_once_or_twice_a_year	144
several_times_a_year	164
about_once_a_month	211
every_week	242
several_times_a_week	89
NA	4

Variable: volunteer_meet_freq

Description: Recent frequency of volunteer work on a seven-point scale.

Question: In the past 12 months, how often did you do volunteer work for religious, charitable, political, health-related, or other organizations?

Table 17

Summary information of the variable volunteer_meet_freq.

	n
never	531
less_than_once_a_year	127
about_once_or_twice_a_year	172
several_times_a_year	170
about_once_a_month	178
every_week	189
several_times_a_week	80
NA	4

Variable: confide_spouse

Description: A derived variable indicating whether a given participant perceived that their social network included a spouse or partner that they could confide in.

Question: From time to time, most people discuss things that are important to them with others. For example, these may include good or bad things that happen to you, problems you are having, or important concerns you may have. Looking back over the last 12 months, how many people do you have in your social network with whom you discussed things that were important to you?

Table 18

Summary information of the variable confide_spouse.

	n
FALSE	430
TRUE	1021
NA	0

Variable: confide_family

Description: A derived continuous variable indicating the number of family members that a given participant perceived to be part of their social network and that they also could confide in.

Question: From time to time, most people discuss things that are important to them with others. For example, these may include good or bad things that happen to you, problems you are having, or important concerns you may have. Looking back over the last 12 months, how many people do you have in your social network with whom you discussed things that were important to you?

Table 19

Summary information of the variable confide_family.

Min	Max	*M*	*SD*	NOBS	NA
0.00	35.00	2.80	3.29	1451	0

Variable: confide_friend

Description: A derived continuous variable indicating the number of friends that a given participant perceived to be part of their social network and that they also could confide in.

Question: From time to time, most people discuss things that are important to them with others. For example, these may include good or bad things that happen to you, problems you are having, or important concerns you may have. Looking back over the last 12 months, how many people do you have in your social network with whom you discussed things that were important to you?

Table 20

Summary information of the variable confide_friend.

Min	Max	*M*	*SD*	NOBS	NA
0.00	35.00	2.80	3.29	1451	0

Variable: confide_colleague

Description: A derived continuous variable indicating the number of colleagues that a given participant perceived to be part of their social network and that they also could confide in.

Question: From time to time, most people discuss things that are important to them with others. For example, these may include good or bad things that happen to you, problems you are having, or important concerns you may have. Looking back over the last 12 months, how many people do you have in your social network with whom you discussed things that were important to you?

Table 21

Summary information of the variable confide_colleague.

Min	Max	*M*	*SD*	NOBS	NA
0.00	35.00	2.80	3.29	1451	0

Variable: num_household

Description: a continuous variable indicating the number of close members of family that also share the household.

Question: How many close members of your social network live in the same household with you?

Table 22

Summary information of the variable num_household.

Min	Max	*M*	*SD*	NOBS	NA
0.00	10.00	0.90	0.91	1451	12

Variable: talk_social_network

Description: Frequency of socializing with the close members of one's social network on a nine-point scale.

Question: How often do you talk to the close members of your social network?

Table 23

Summary information of the variable talk_social_network.

	n
never	21
less_than_once_a_year	8
once_a_year	2
a_couple_times_a_year	13
once_a_month	38
once_every_two_weeks	72
once_a_week	140
several_times_a_week	396
every_day	759
NA	2

Variable: divulge_family

Description: Self-assessed frequency of being able to discuss with family members about important matters on a four-point scale.

Question: How often can you open up to members of your family?

Table 24

Summary information of the variable divulge_family.

	n
never	83
hardly_ever	168
some_of_the_time	457
often	736
NA	7

Variable: rely_family

Description: Self-assessed frequency of being able to rely on family members for important matters on a four-point scale.

Question: How often can you rely on members of your family?

Table 25

Summary information of the variable rely_family.

	n
never	76
hardly_ever	119
some_of_the_time	398
often	851
NA	7

Variable: divulge_friend

Description: Self-assessed frequency of being able to discuss with friends about important matters on a four-point scale.

Question: How often can you open up to your friends?

Table 26

Summary information of the variable divulge_friend.

	n
never	83
hardly_ever	159
some_of_the_time	652
often	548
NA	9

Variable: rely_friend

Description: Self-assessed frequency of being able to rely on friends for important matters on a four-point scale.

Question: How often can you rely on your friends?

Table 27

Summary information of the variable rely_friend.

	n
never	69
hardly_ever	125
some_of_the_time	616
often	632
NA	9

Variable: divulge_spouse

Description: Self-assessed frequency of being able to rely on spouse/partner for important matters on a four-point scale.

Question: How often can you open up to your spouse or partner?

Table 28

Summary information of the variable divulge_spouse.

	n
never	440
hardly_ever	61
some_of_the_time	195
often	735
NA	20

Variable: rely_spouse

Description: Self-assessed frequency of being able to rely on spouse or partner for important matters on a four-point scale.

Question: How often can you rely on your spouse or partner?

Table 29

Summary information of the variable rely_spouse.

	n
never	427
hardly_ever	42
some_of_the_time	165
often	794
NA	23

Variable: health

Description: Self-assessed health status on a four-point scale.

Question: All in all, would you say that your health is generally…

Table 30

Summary information of the variable health.

	n
poor	63
fair	302
good	829
excellent	254
NA	3

Variable: learning_disability

Description: Self-reported previous learning disability.

Question: Have you ever been diagnosed with a learning disability?

Table 31

Summary information of the variable learning_disability.

	n
no	1397
yes	50
NA	4

Variable: employed

Description: Self-reported employment status: not employed (no), fully employed (full) or employed part-time (part).

Question: If you are not retired, are you currently working for pay?

Table 32

Summary information of the variable employed.

	n
full	444
no	673
part	328
NA	6

Variable: volunteer

Description: Self-reported volunteer work on a three-point scale: full time (full), part time (part) and not doing volunteer work (no).

Question: Are you currently doing unpaid work?

Table 33

Summary information of the variable volunteer.

	n
full	23
no	1234
part	188
NA	6

Variable: living_arrangements

Description: Self-reported living arrangement with three answer options.

Question: What are your current living arrangements?

Table 34

Summary information of the variable living_arrangements

	n
alone	372
else	244
spouse_partner	833
NA	2

Variable: residency

Description: Self-reported description of one's current residency with three answer options.

Question: Please describe your living location? I live in a...

Table 35

Summary information of the variable residency

	n
nursing_home	2
private_apartment_or_home	1420
residential_care_or_home	24
NA	5

Variable: testing_session

Description: A derived and discretized variable indicating the time point of the testing session. The variable consists of the following five levels:

- t1 = 2019
- t2 = from 2020-04-08 to 2020-06-16
- t3 = from 2020-06-17 to 2020-06-30
- t4 = from 2020-10-14 to 2020-11-05
- t5 = from 2021-01-12 to 2021-02-15

Table 36

Summary information of the variable testing_session

	n
t1	455
t2	256
t3	408
t4	162
t5	170
NA	0

Variable: broadcast_habit

Description: Self-reported frequency of consuming broadcast programming.

Question: How many hours a day do you watch broadcast programming (TV, Internet) or listen to the radio?

Table 37

Summary information of the variable broadcast_habit.

Min	Max	*M*	*SD*	NOBS	NA
0.00	12.00	2.94	2.21	1451	28

Variable: country

Description: A derived variable from the self-reported current country of residency.

Question: Which state/province do you currently live in?

Table 38

Summary information of the variable country

	n
Canada	71
USA	1369
NA	11

Variable: state_province

Description: A derived variable from the self-reported current country of residency.

Question: Which state/province do you currently live in?

Table 39

Summary information of the variable state_province.

	n
alabama	17
alaska	2
alberta	7
arizona	42
arkansas	18
britishcolumbia	9
california	117
colorado	21
connecticut	12
delaware	4
districtofcolumbia	1
florida	141
georgia	54
hawaii	4
idaho	6
illinois	51
indiana	28
iowa	11
kansas	7
kentucky	28
louisiana	10

maine	3
manitoba	2
maryland	15
massachusetts	27
michigan	53
minnesota	16
mississippi	9
missouri	30
montana	2
nebraska	5
nevada	11
newbrunswick	2
newhampshire	5
newjersey	36
newmexico	6
newyork	70
newfoundland	1
northcarolina	64
northdakota	2
novascotia	4
ohio	59
oklahoma	18
ontario	35
oregon	28
pennsylvania	50
quebec	5
rhodeisland	5
saskatchewan	5

southcarolina	20
southdakota	3
tennessee	29
texas	92
utah	7
vermont	6
virginia	41
washington	45
westvirginia	8
wisconsin	30
yukonterritory	1
NA	11

CoSoWELL: Corpus

Variable: id

Description: A unique identifier of the participants (N = 1,178). This variable can be used to link the corpus data of a given participant with their survey, when available (n = 1028).

Variable: doc_id

Description: A unique identifier of the written narratives (N = 7,975).

Variable: paragraph_id

Description: An index number of a paragraph in a given narrative in a linear order starting from 1. This variable is only provided as part of the output of the parser as the narratives were not broken down into separate paragraphs during parsing.

Variable: sentence_id

Description: An index number of a sentence in a given narrative in a linear order starting from 1.

Table 40

Summary information of the variable sentence_id.

Min	Max	*M*	*SD*	NOBS	NA
1	92	7.59	7.15	74815	0

Variable: sentence

Description: A sentence where a given token is realized.

Variable: token_id

Description: An index number of a token in a given sentence in a linear order starting from 1.

Table 41

Summary information of the variable token_id.

Min	Max	*M*	*SD*	NOBS	NA
1	99		10.43	1338320	0

Variable: token

Description: A lexical realization of a word in a given sentence.

Variable: lemma

Description: A lemma of a given token.

Variable: upos

Description: A universal part-of-speech tag of a given token.

Table 42

Summary information of the variable upos.

	n
ADJ	71178
ADP	119213
ADV	85526
AUX	88701
CCONJ	52511
DET	111594
INTJ	1645
NOUN	224681
NUM	14185
PART	41290
PRON	175937
PROPN	18526
PUNCT	127155
SCONJ	26515
SYM	565
VERB	178662
X	436
NA	0

Variable: xpos

Description: A Stanford part-of-speech tag of a given token.

	n
XLRB	1234
XRRB	1226
X	43440
X1	258
	74039
X	2430
X1	2320
X2	175
ADD	37
AFX	153
CC	52512
CD	14185
DT	114429
EX	2026
FW	166
GW	61
HYPH	2179
IN	136672
JJ	66241
JJR	3037
JJS	1898
LS	19
MD	17968
NFP	92
NN	181309

NNP	18102
NNPS	427
NNS	47316
PDT	1587
POS	2419
PRP	116439
PRP	38731
RB	85449
RBR	1793
RBS	847
RP	9069
SYM	244
TO	28079
UH	1645
VB	58681
VBD	86537
VBG	33433
VBN	24012
VBP	25878
VBZ	20858
WDT	5687
WP	4726
WP	42
WRB	8213
NA	0

Variable: feats

Description: Morphological features of a given token. The morphological tags are separated by a | symbol.

	n
AbbrYes	83
AbbrYesMoodIndTensePresVerbFormFin	3
AbbrYesNumberPlur	2
AbbrYesNumberSing	3
AbbrYesPronTypeInt	1
AbbrYesTensePresVerbFormPart	10
CaseAccGenderFemNumberSingPerson3PronTypePrs	2502
CaseAccGenderFemNumberSingPerson3PronTypePrsReflexYes	200
CaseAccGenderMascNumberSingPerson3PronTypePrs	2745
CaseAccGenderMascNumberSingPerson3PronTypePrsReflexYes	182
CaseAccGenderNeutNumberSingPerson3PronTypePrs	4064
CaseAccGenderNeutNumberSingPerson3PronTypePrsReflexYes	33
CaseAccNumberPlurPerson1PronTypePrs	1870
CaseAccNumberPlurPerson1PronTypePrsReflexYes	85
CaseAccNumberPlurPerson3PronTypePrs	2421
CaseAccNumberPlurPerson3PronTypePrsReflexYes	137
CaseAccNumberSingPerson1PronTypePrs	7342
CaseAccNumberSingPerson1PronTypePrsReflexYes	783
CaseAccNumberSingPerson2PronTypePrsReflexYes	30
CaseAccPerson2PronTypePrs	332
CaseNomGenderFemNumberSingPerson3PronTypePrs	9412
CaseNomGenderMascNumberSingPerson3PronTypePrs	7490
CaseNomGenderNeutNumberSingPerson3PronTypePrs	10058
CaseNomGenderNeutNumberSingPerson3PronTypePrsTypoYes	4

CaseNomNumberPlurPerson1PronTypePrs	11443
CaseNomNumberPlurPerson3PronTypePrs	4241
CaseNomNumberPlurPerson3PronTypePrsTypoYes	1
CaseNomNumberSingPerson1PronTypePrs	49039
CaseNomPerson2PronTypePrs	1430
DefiniteDefPronTypeArt	58239
DefiniteIndPronTypeArt	34317
DegreeCmp	4122
DegreePos	67645
DegreePosNumTypeOrd	1704
DegreePosTypoYes	16
DegreeSup	2319
ForeignYes	18
GenderFemNumberSingPerson3PossYesPronTypePrs	5697
GenderFemNumberSingPerson3PronTypePrs	1
GenderMascNumberSingPerson3PossYesPronTypePrs	3711
GenderMascNumberSingPerson3PronTypePrs	63
GenderNeutNumberSingPerson3PossYesPronTypePrs	168
GenderNeutNumberSingPerson3PronTypePrs	18
MoodImpVerbFormFin	1599
MoodImpVerbFormInf	3
MoodIndNumberSingPerson1TensePastVerbFormFin	2065
MoodIndNumberSingPerson1TensePresVerbFormFin	2451
MoodIndNumberSingPerson3TensePastVerbFormFin	21165
MoodIndNumberSingPerson3TensePresVerbFormFin	20858
MoodIndNumberSingTensePastVerbFormFin	23
MoodIndTensePastTypoYesVerbFormFin	21
MoodIndTensePastVerbFormFin	63263

MoodIndTensePresTypoYesVerbFormFin	3
MoodIndTensePresVerbFormFin	23421
NumberPlur	47730
NumberPlurPerson1PossYesPronTypePrs	3763
NumberPlurPerson1PossYesPronTypePrsTypoYes	2
NumberPlurPerson1PronTypePrs	1
NumberPlurPerson3PossYesPronTypePrs	1860
NumberPlurPerson3PronTypePrs	12
NumberPlurPronTypeDem	1244
NumberPlurTypoYes	11
NumberSing	199385
NumberSingPerson1PossYesPronTypePrs	23082
NumberSingPerson1PronTypePrs	111
NumberSingPerson2PronTypePrs	1
NumberSingPronTypeDem	9962
NumberSingTypoYes	27
NumTypeCard	14185
NumTypeMult	420
Person2PossYesPronTypePrs	431
PossYesPronTypeInt	42
PronTypeDem	4764
PronTypeInt	10977
PronTypeRel	7616
TensePastTypoYesVerbFormPart	1
TensePastTypoYesVerbFormPartVoicePass	4
TensePastVerbFormPart	16823
TensePastVerbFormPartVoicePass	7184
TensePresVerbFormPart	11999

TypoYes	78
TypoYesVerbFormFin	12
TypoYesVerbFormGer	13
TypoYesVerbFormInf	8
VerbFormFin	17956
VerbFormGer	21407
VerbFormInf	57071
NA	451282

Variable: head_token_id

Description: An index number of a given head for each token in a sentence. If the token is the root of the sentence, this index is zero.

Variable: dep__rel

Description: A universal dependency relation between a given token and its head.

	n
acl	8958
aclrelcl	14739
advcl	30985
advmod	87337
amod	46901
appos	2308
aux	46762
auxpass	7549
case	110706
cc	52491
ccpreconj	345
ccomp	17635
compound	26014
compoundprt	9158
conj	57623
cop	33764
csubj	889
det	107829
detpredet	1684
discourse	1475
expl	2768
fixed	2668
flat	1005
goeswith	112
iobj	1683

list	46
mark	58962
nmod	30214
nmodnpmod	293
nmodposs	40374
nmodtmod	183
nsubj	131508
nsubjpass	6191
nummod	10240
obj	75061
obl	69469
oblnpmod	3159
obltmod	4663
orphan	1
parataxis	5183
punct	126781
reparandum	4
root	74815
vocative	162
xcomp	27623
NA	0

Variable: deps

Description: Provided as part of the output of the parser.

Variable: misc

Description: Provided as part of the output of the parser.

Variable: worktimeinseconds

Description: The total time spent on the writing task measured in seconds.

Table 46

Summary information of the variable worktimeinseconds.

Min	Max	*M*	*SD*	NOBS	NA
51.76	14188.70	2054.82	1497.76	7975	0

Variable: narrative

Description: A written narrative produced by a given participant in the writing task.

Variable: type

Description: A type of the prompt used to elicit a given written narrative.

Table 47

Summary information of the variable type

	n
cookie	1993
future	1994
past	1994
yesterday	1994
NA	0

Variable: testing_session

Description: A derived and discretized variable indicating the time point of the testing session. The variable consists of the following five levels:

- t1 = 2019
- t2 = from 2020-04-08 to 2020-06-16
- t3 = from 2020-06-17 to 2020-06-30
- t4 = from 2020-10-14 to 2020-11-05
- t5 = from 2021-01-12 to 2021-02-15

Table 48

Summary information of the variable testing_session

	n
t1	848
t2	2111
t3	1708
t4	1640
t5	1668
NA	0
