# Supplementary material for: Cognitive and social well-being in older adulthood: The CoSoWELL corpus of written life stories
Source: Behav Res Methods. 2022 Aug 24;55(6):2885–909. doi: 10.3758/s13428-022-01926-0 (PMC9400578; doi:10.3758/s13428-022-01926-0)
Supplement: Supplementary file 2 — (PDF 236 KB) [file 13428_2022_1926_MOESM2_ESM.pdf]

Supplementary material

Aki-Juhani Kyröläinen

McMaster University and Brock University

James Gillett

McMaster University

Megan Karabin

McMaster University

Ranil Sonnadara

McMaster University and Vector Institute

Victor Kuperman

McMaster University

Supplementary material

Supplementary material S1: Lexico-syntactic profile and story type

In this section, we explore linguistic variation across story types, with a focus on lexico-syntactic variables. The overarching goal of this analysis is to determine whether differences in story types—presumably related to different facets of autobiographical memories and ideation—manifest themselves in formal linguistic properties of the narratives.

The first variable considered here is Noun-to-Verb Ratio, calculated by dividing the number of nouns by the number of verbs used in a given narrative ($\frac{tokens_{noun}}{tokens_{noun}+tokens_{verb}}$). The importance of this variable stems from the fact that distributional properties of nouns and verbs affect language processing in general (see Vigliocco et al., 2011). Moreover, differences in production and comprehension of nouns and verbs are associated with language impairments that increase in their incidence with advancing age, such as dementia and Alzheimer's disease. Both types of impairments are associated with telegraphic style characterized by a less frequent use of verbs relative to nouns, i.e., higher Noun-to-Verb ratio (Eyigoz et al., 2020; Le et al., 2011; Burke & Shafto, 2008).

A second set of variables under consideration provides several estimates of Type-Token Ratio (TTR) originally proposed by Johnson (1944) as a metric of lexical diversity in a language. Traditionally, TTR is calculated as the number of word types in a text divided by the number of word tokens but other operationalizations are possible (for discussion see Kyle, 2019). In this study, we employed three related but different operationalizations of this metric: 1) based on words in line with the traditional definition ($\frac{types_{word}}{tokens_{word}}$, labeled here TTR-w), 2) based on the part-of-speech associated with a given word ($\frac{types_{pos}}{tokens_{pos}}$, labeled TTR-p) and, finally, 3) based on the dependency relation associated with a particular word ($\frac{types_{dep}}{tokens_{dep}}$, TTR-d).[1] An increase in any of the three TTR measures can be interpreted as an increase in lexical (TTR-w) or syntactic

[1] In the calculation of TTR-w, punctuation was excluded.

diversity (TTR-p and TTR-d), signaling a greater linguistic productivity of an individual writer in a given narrative.

A third set of variables tapped into the syntactic complexity of a sentence that has been shown to affect language comprehension (e.g., Gibson, 2000; Gibson, 1998; Levy, 2008) and constrain language production (Ferreira, 1991; Scontras et al., 2015; Szmrecsanyi, 2004; see Nippold et al., 2014, for results across age groups). The first variable in this set captured how elaborate the syntactic constructions were in a given sentence, i.e., it calculates how many syntactic dependencies are there in a sentence relative to the total number of words in that sentence. Since each dependency has exactly one head, the formula for the measure that we will refer to as d-Ratio was: $1 - \frac{n_{head}}{n_{token}}$. A higher d-Ratio indicates that a sentence contains more dependencies relative to its number of tokens, making the sentence syntactically more elaborate. This measure was calculated for every sentence and then averaged for each narrative.

The second variable in this set operationalized syntactic complexity in a given sentence by calculating the longest path in a dependency tree for the sentence. For example, in the sentence visualized in Figure 1, the longest dependency path was 3. This measure follows the notion that longer dependency relations are more effortful to process (Gibson, 2000; Gibson, 1998) and there is a general tendency to minimize dependency distances in language production (Futrell et al., 2015; Temperley, 2007). To facilitate efficient computation time, a given dependency tree was treated as a directed acyclic graph (see Oya, 2011; Yadav et al., 2019) and the longest path was calculated using the diameter function in the R package igraph, version 1.2.6, i.e., the length of the longest shortest path ($max_{u,v}d(u, v)$ between any two nodes (u, v), where $d(u, v)$ is a distance (Csardi, Nepusz, et al., 2006). Similar to d-Ratio, this variable was calculated for each sentence and averaged across the sentences for a given narrative. We will refer to this variable as the longest dependency path (LDP) in this study.

The third and final variable in this set considered the notion of syntactic complexity at the level of the narrative by focusing on the use of complex clauses such as coordination and subordination relative to syntactically simplex ones (e.g., Beaman,

1984; Givón, 1991). For the purposes of the present study, a wide range of syntactic constructions were considered to constitute degree of embeddedness, not just coordinating and subordinating clauses. The presence of one of the following nine dependency relations was used to mark a sentence as complex listed below, otherwise a sentence was considered as simplex. The definition used below follows the UD schema used in this study.

- parataxis: parataxis
- xcomp: a clausal complement of a verb or an adjective functioning as a predicative or clausal complement without its own subject
- ccomp: a clausal complement of a verb or adjective with a dependent clause which is a core argument
- advcl: an adverbial clause modifier
- acl:relcl: a relative clause modifier
- acl: finite and non-finite clauses modifying a nominal
- conj: a coordinating conjunction
- cc: a conjunct and a preceding coordinating conjunction
- mark: a word marking a clause as subordinate to another clause

For a given narrative, the number of simplex and complex sentences calculated and the sums were divided to compute a ratio $(1 - \frac{n_{simplex}}{n_{complex}})$ where higher values indicated that a given story had relatively more complex sentences.

The final variable considered in this study is mean length of utterance (MLU) originally proposed by Brown (1973). MLU has been used extensively to study linguistic productions in children (see Rice et al., 2010, and citations therein) and adults (see Nippold et al., 2014, and citations therein). MLU can be operationalized either based on the number of morphemes or words (for recent discussion see Ezeizabarrena & Garcia Fernandez, 2018). Here, we opted for the simpler word-based calculation $(\frac{n_{word}}{n_{sentence}})$.

A total of eight stories were removed from the data due to undefined values in calculating the eight lexico-syntactic variables. Thus, the data used in this section consisted of 7,275 life stories written by 1,028 participants. The summary information of the lexico-syntactic variables across the story types is given in Table 1.

Table 1

Descriptive statistics of the lexico-syntactic profile of the four story types.

	Story type							
	Cookie		Future		Past		Yesterday	
Variable	*M*	*SD*	*M*	*SD*	*M*	*SD*	*M*	*SD*
Noun-to-Verb Ratio	0.56	0.09	0.56	0.08	0.56	0.08	0.56	0.09
TTR-d	0.22	0.11	0.23	0.13	0.16	0.09	0.21	0.12
TTR-p	0.12	0.08	0.13	0.09	0.09	0.06	0.12	0.08
TTR-w	0.63	0.1	0.63	0.1	0.56	0.09	0.63	0.11
d-Ratio	0.59	0.04	0.6	0.04	0.59	0.03	0.59	0.04
LDP	4.08	1.27	4.11	1.16	3.95	1.06	4	1.12
Embeddedness	0.76	0.3	0.76	0.34	0.76	0.31	0.74	0.36
MLU	19.41	8.8	19.65	7.98	18.91	8.03	18.82	8.51

To analyze the potential differences in the lexico-syntactic profile of the story types, we fitted eight separate linear mixed-effects model to the data. The package lme4 version 1.1-26 was used to fit the mixed-effects models (Bates et al., 2015) and the *p*-values and ANOVA type III were estimated using Satterthwaite's degrees of freedom method as implemented in the R package lmerTest (version 3.1-3) (Kuznetsova et al., 2017). In a given model, the lexico-syntactic response variable was modeled as a function of the story type and participants were included in the model as random intercepts.

Figure 1 visualizes the estimated effect of story type on each of the eight lexico-syntactic response variables. Overall, regression models demonstrated that story types come with distinctive lexico-syntactic profiles, since noun-to-verb ratio and

embededness was the only response variables under consideration that did not give rise to statistically significant contrasts between the story types.

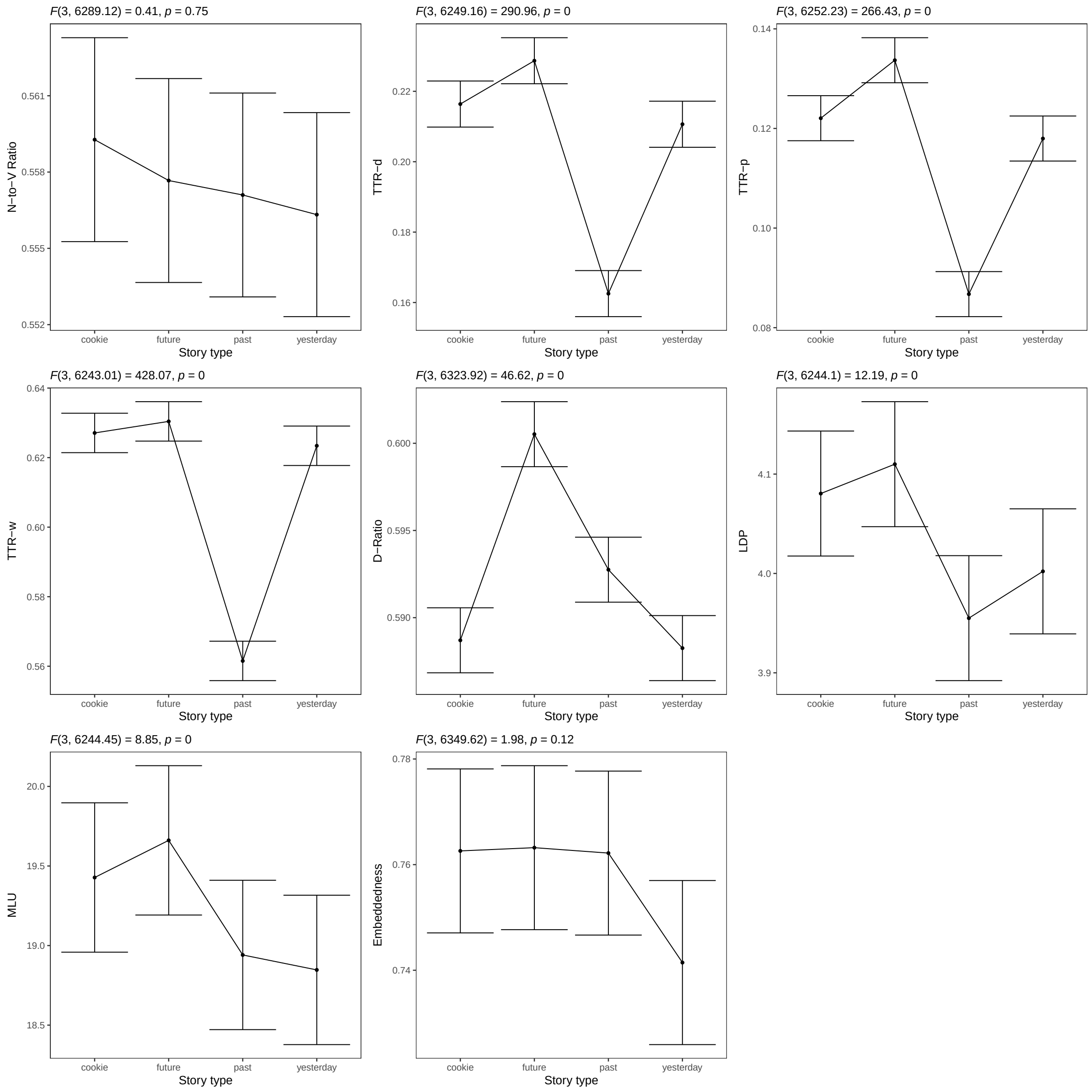

Figure 1. Estimated effect of story type on lexico-syntactic response variables. Error bars represent 95% CIs.

The profile of story type "future" was associated with a relatively high lexical and syntactic diversity as indicated by the type-token ratios in Figure 1) and increased syntactic elaborateness (see d-Ratio in Figure 1). Furthermore, stories about the future tended to be syntactically more complex (see LDP in Figure 1) and also longer (see MLU in in Figure 1). Story type "past" had a similarly distinctive profile. Stories about

distant past were associated with decreased lexical (see TTR-w) and syntactic (see TTR-p and TTR-d) richness. They also tended to be shorter than the future stories (see MLU in Figure 1) and characterized by syntactic simplicity (see LDP in Figure 1). Stories about the present (story type "yesterday") displayed a similar profile to stories about the past with one crucial difference, namely, they were much richer in terms of both lexical (see TTR-w) and syntactical diversity (see TTR-p and TTR-d). Finally, "cookie" as a story type displayed a profile similar to "future" except for a relatively weak syntactic elaborateness (d-Ratio).

In sum, the analysis presented in this section demonstrated that story types displayed robust differences in their lexico-syntactic profiles. Interestingly, stories associated with past events appeared to be less vivid both lexically and syntactically and this lack of elaborateness increased the further back in veridical time the depicted life story was situated in. In contrast, the lexico-syntanctic profile of the future story type was characterized by highly elaborate use of lexical and syntactic devices. Finally, the results presented in this section demonstrated that the story types used in this study tap into different language patterns, providing a method that brings forth a comprehensive snapshot of an individual's language use and personal life experiences.

Supplementary material S2: Data generating process of STM

The data-generating process of STM is shown graphically in Figure 2. Conceptually, the model consists of three parts: 1) a prevalence model controlling the allocation of words to topics as a function of covariates, 2) a topical content model controlling for the frequency of each words in a given topic as a function of covariates, and 3) a language model combining these two sources to generate the actual words in each document (for a detailed description of the model, we refer the reader to Roberts et al., 2019). Algorithmically, this type of topic model combines properties of the correlated topic model (CTM) (Blei & Lafferty, 2007), the Dirichlet-Multinomial Regression (DMR) topic model (Mimno & McCallum, 2008) and the Sparse Additive Generative (SAGE) topic model (Eisenstein et al., 2011). The structural topic model

(STM) used here is implemented in the R package stm, version 1.3.6, (Roberts et al., 2019).

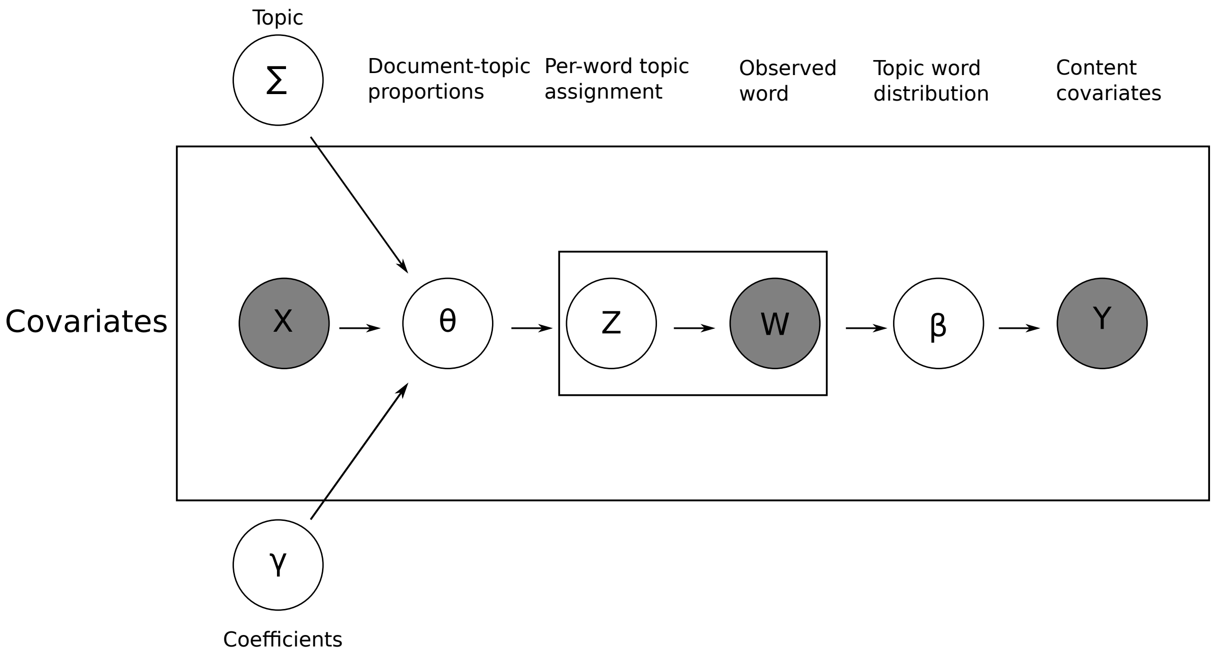

Figure 2. A graphical illustration of the structural topic model adapted from Roberts et al. (2016).

Supplementary material S3: Example of broad and narrow document-topic distribution

We illustrate the ability of a topic model to associate a specific narrative with a document-topic distribution. We took two extreme cases as examples in the story type "yesterday" (recent past): a narrative that was estimated to be dominated by a single topic while the second story was estimated to display multiple topics simultaneously. The former story is given as Example 1 and its document-topic distribution is visualized as a line graph in Figure 3 (top panel) where the x-axis contains the topics and the y-axis contains the document-topic probability (which sums to one for a given narrative). The changes in the document-topic distribution are visualized as a line.

> Example 1: I went grocery shopping yesterday at two stores. The first one did not require face masks anymore, but the other one did. I must admit I

felt safer in the second store and probably only shop there until the other one goes back to requiring masks. Got all the stuff I needed and walked home. Great exercise. A total of almost 3 miles walking and carrying groceries.

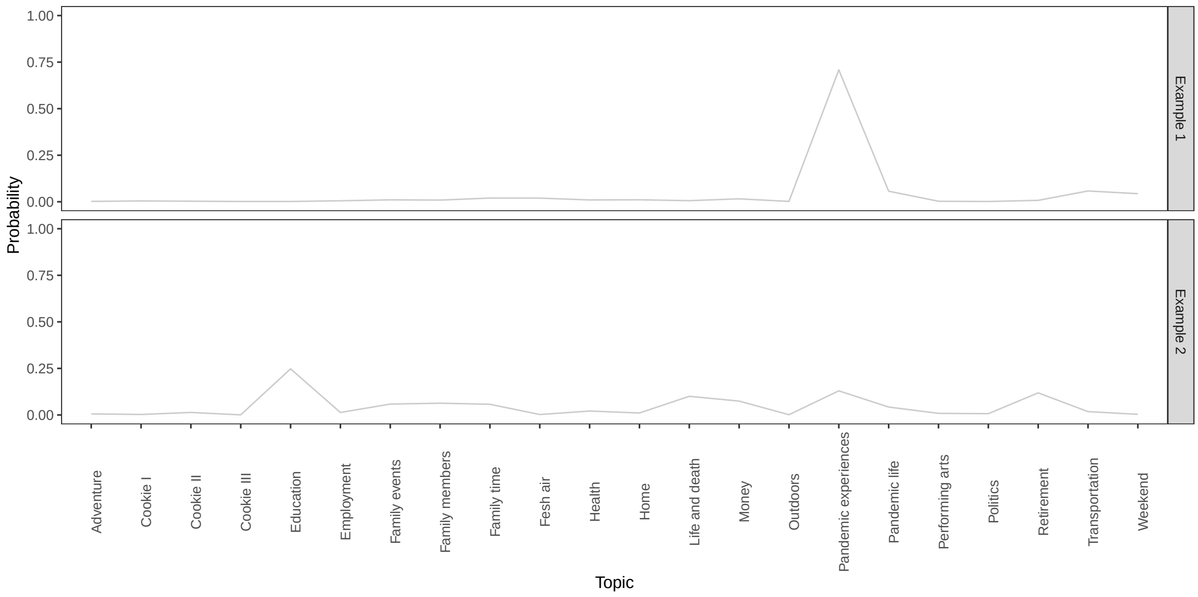

Figure 3. Estimated topic distribution across two life stories.

Example 1 was estimated to be primarily associated with a single topic, namely PANDEMIC EXPERIENCES. Based on a close reading of the narrative, it is clear that the story described an event related to COVID-19. The fitted STM was able construct and identify this abstract topic and associate it strongly with this particular narrative.

Example 2, written about a future event, illustrates the opposite pattern—a mixture of multiple topics. Its topic distribution is visualized in Figure 3 (bottom panel). The estimated topic distribution reveals that this particular story does not demonstrate a well-defined topic structure as it pertains lexical elements from such topics as EDUCATION, LIFE AND DEATH, PANDEMIC EXPERIENCES and RETIREMENT. While the narrative can be seen as lacking in cohesiveness (Halliday & Hasan, 1976), the key elements verbalized in the narrative were well captured with STM.

Example 2: Since the advent of COVID 19, I have isolated due to my multiple health risks. One of my granddaughters tested positive in

December, so I would call her nightly to check on her. She never developed symptoms and it is possible that she had a false positive as no one else in the family ever tested positive. So, in last night's call, I asked her about some concerns I had. Not concerns so much about COVID, but concerns about other issues in our society. I asked her if she knew of anyone who engaged in "cutting". She thought that meant their wrists. I explained that it was generally in places hidden by clothing. I asked her if she had ever heard of anyone talking about suicide. Or bringing a gun to school. Or being bullied. I asked her if she had someone, at school, that she would trust to confide in...teacher, coach, bus driver...in case it ever became relevant. Recently, a 10 year old had brought a loaded gun to school in the town between us. He was being bullied. If she heard about any of these things, would she know who to turn to? It made her think. She mentally ran through her teachers and named one that she would feel comfortable to confide. I told her about an incident, years ago, where a girl told my daughter about things that were happening to her. I reached out to a teacher that I volunteered for and told her my concerns. I wanted the information to get to people that could help this girl. I feel I did the right thing. I just wanted my granddaughter to have an idea what to do, similar to a fire drill, so she was prepared.

Supplementary material S4: Global variable importance

As a measure of variable importance, we used the permutation variable importance Breiman (2001). This metric is based on the idea of breaking the original association between a given predictor and the response by randomly permuting said predictor. If the permuted predictor was important, the model performance will decrease when predictions are made with the permuted variable. The difference in the model performance before and after permutation is then used as a measure of variable importance. The permutation importance was computed for the 22 predictors and their relative ranking is visualized in Figure 4.

The cookie-related predictors (cookie I, cookie II, and cookie III) were estimated

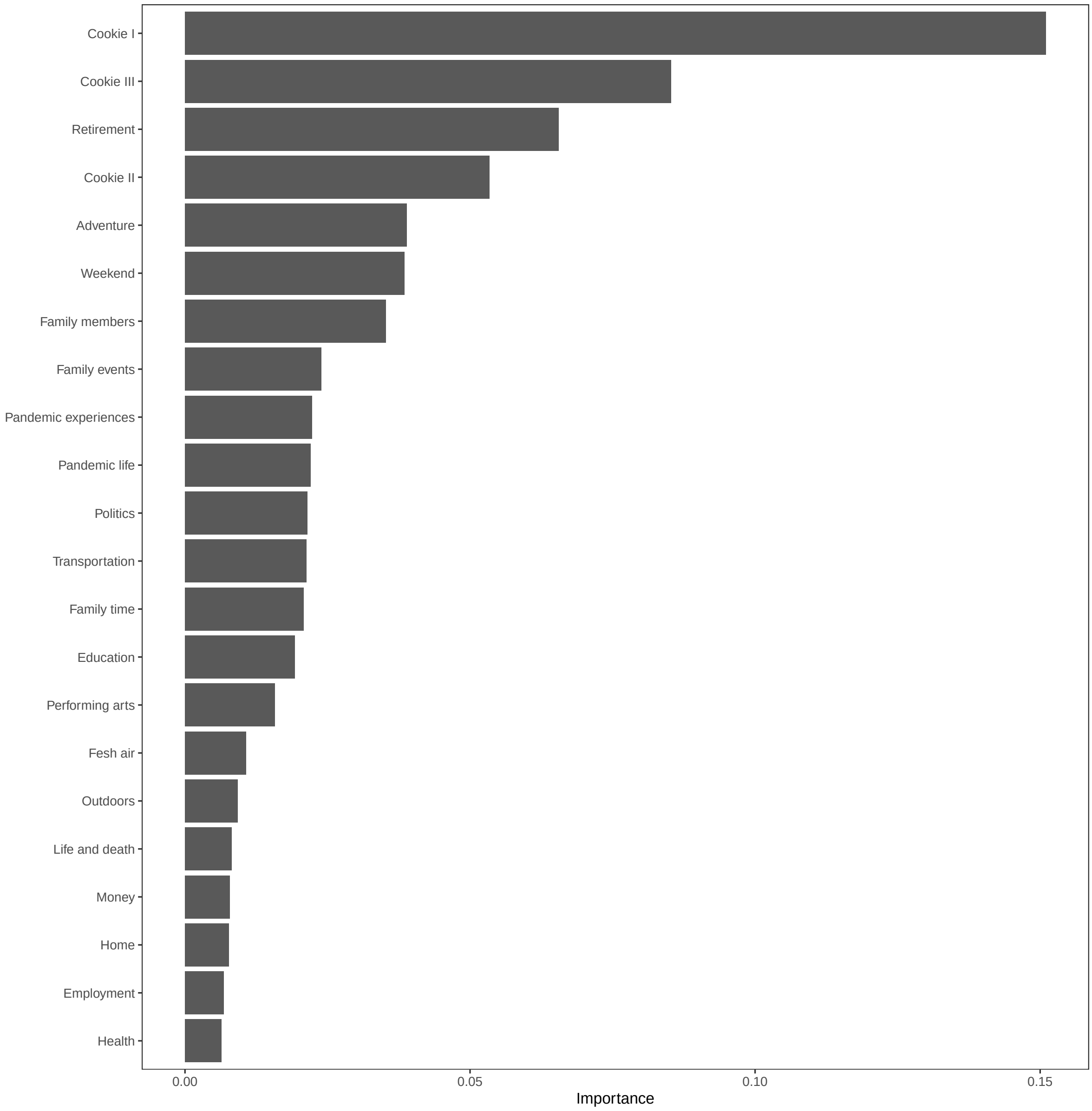

Figure 4. Relative variable importance in classifying the four story types.

to be highly important. This is to be expected given that as a type the cookie stories were extremely well discriminated. More importantly, the relative ranking of the predictors indicated that predictors that could be linked to autobiographical memories were z estimated to have provided a significant contribution to the model performance, for example retirement, weekend, and the family-related predictors such as family members. It is also noteworthy that variables related to the current COVID-19 crisis were estimated to be important, suggesting that people wrote about important but recent life events. Thus, the estimated variable importance suggests that the

discrimination of the story types might, at least partly, be based on groupings of predictors that can be linked to autobiographical memories. In order to investigate this possibility, the contribution of an individual predictor in discriminating a given story type was estimated.

References

Bates, D., Mächler, M., Bolker, B., & Walker, S. (2015). Fitting linear mixed-effects models using lme4. *Journal of Statistical Software*, *67*(1), 1–48. https://doi.org/10.18637/jss.v067.i01

Beaman, K. (1984). Coordination and subordination revisited: Syntactic complexity in spoken and written narrative discourse. In D. Tannen (Ed.), *Coherence in spoken and written discourse* (pp. 45–80). Ablex.

Blei, D. M., & Lafferty, J. D. (2007). A correlated topic model of science. *The Annals of Applied Statistics*, *1*(1), 17–35.

Breiman, L. (2001). Random forests. *Machine Learning*, *45*(1), 5–32.

Brown, R. (1973). Development of the first language in the human species. *American Psychologist*, *28*(2), 97–106. https://doi.org/10.1037/h0034209

Burke, D. M., & Shafto, M. A. (2008). Language and aging. In F. I. M. Craik & T. A. Salthouse (Eds.), *The handbook of aging and cognition* (pp. 373–443). Psychology Press.

Csardi, G., Nepusz, T. et al. (2006). The igraph software package for complex network research. *InterJournal, Complex Systems*, *1695*(5), 1–9.

Eisenstein, J., Ahmed, A., & Xing, E. P. (2011). *Sparse additive generative models of text.*

Eyigoz, E., Mathur, S., Santamaria, M., Cecchi, G., & Naylor, M. (2020). Linguistic markers predict onset of Alzheimer's disease. *EClinicalMedicine*, *28*, 100583. https://doi.org/10.1016/j.eclinm.2020.100583

Ezeizabarrena, M.-J., & Garcia Fernandez, I. (2018). Length of utterance, in morphemes or in words?: Mlu3-w, a reliable measure of language development in early basque. *Frontiers in Psychology*, *8*, 2265. https://doi.org/10.3389/fpsyg.2017.02265

Ferreira, F. (1991). Effects of length and syntactic complexity on initiation times for prepared utterances. *Journal of Memory and Language*, *30*(2), 210–233. https://doi.org/10.1016/0749-596x(91)90004-4

Futrell, R., Mahowald, K., & Gibson, E. (2015). Large-scale evidence of dependency length minimization in 37 languages. *Proceedings of the National Academy of Sciences*, *112*(33), 10336–10341. https://doi.org/10.1073/pnas.1502134112

Gibson, E. (1998). Linguistic complexity: Locality of syntactic dependencies. *Cognition*, *68*(1), 1–76. https://doi.org/10.1016/S0010-0277(98)00034-1

Gibson, E. (2000). The dependency locality theory: A distance-based theory of linguistic complexity. In A. Marantz, Y. Miyashita, & W. O'Neil (Eds.), *Image, language, brain: Papers from the first mind articulation project symposium* (pp. 95–126). The MIT Press.

Givón, T. (1991). Markedness in grammar: Distributional, communicative and cognitive correlates of syntactic structure. *Studies in Language*, *15*(2), 335–370.

Halliday, M. A. K., & Hasan, R. (1976). *Cohesion in english*. Longman.

Johnson, W. (1944). Studies in language behavior: I. A program of research. *Psychological Monographs*, *56*, 1–15.

Kuznetsova, A., Brockhoff, P. B., & Christensen, R. H. B. (2017). lmerTest package: Tests in linear mixed effects models. *Journal of Statistical Software*, *82*(13), 1–26. https://doi.org/10.18637/jss.v082.i13

Kyle, K. (2019). Measuring lexical richness. In S. Webb (Ed.), *The routledge handbook of vocabulary studies* (pp. 454–475). Routledge.

Le, X., Lancashire, I., Hirst, G., & Jokel, R. (2011). Longitudinal detection of dementia through lexical and syntactic changes in writing: A case study of three British novelists. *Literary and linguistic computing*, *26*(4), 435–461. https://doi.org/10.1093/llc/fqr013

Levy, R. (2008). Expectation-based syntactic comprehension. *Cognition*, *106*(3), 1126–1177. https://doi.org/10.1016/j.cognition.2007.05.006

Mimno, D. M., & McCallum, A. (2008). Topic models conditioned on arbitrary features with dirichlet-multinomial regression. *Proceedings of the Twenty-Fourth Conference on Uncertainty in Artificial Intelligence*, *24*, 411–418.

Nippold, M. A., Cramond, P. M., & Hayward-Mayhew, C. (2014). Spoken language production in adults: Examining age-related differences in syntactic complexity. *Clinical Linguistics & Phonetics*, *28*(3), 195–207. https://doi.org/10.3109/02699206.2013.841292

Oya, M. (2011). Syntactic dependency distance as sentence complexity measure. *Proceedings of the 16th International Conference of Pan-Pacific Association of Applied Linguistics*, 313–316.

Rice, M. L., Smolik, F., Perpich, D., Thompson, T., Rytting, N., & Blossom, M. (2010). Mean length of utterance levels in 6-month intervals for children 3 to 9 years with and without language impairments. *Journal of Speech, Language & Hearing Research*, *53*(2), 333–349. https://doi.org/10.1044/1092-4388(2009/08-0183

Roberts, M. E., Stewart, B. M., & Airoldi, E. M. (2016). A model of text for experimentation in the social sciences. *Journal of the American Statistical Association*, *111*(515), 988–1003. https://doi.org/10.1080/01621459.2016.1141684

Roberts, M. E., Stewart, B. M., & Tingley, D. (2019). stm: An R package for structural topic models. *Journal of Statistical Software*, *91*(2), 1–40. https://doi.org/10.18637/jss.v091.i02

Scontras, G., Badecker, W., Shank, L., Lim, E., & Fedorenko, E. (2015). Syntactic complexity effects in sentence production. *Cognitive Science*, *39*(3), 559–583. https://doi.org/10.1111/cogs.12168

Szmrecsanyi, B. M. (2004). On operationalizing syntactic complexity. In G. Purnelle, C. Fairon, & A. Dister (Eds.), *Proceedings of the 7th international conference on textual data statistical analysis* (pp. 1032–1039). Presses universitaires de Louvain.

Temperley, D. (2007). Minimization of dependency length in written English. *Cognition*, *105*(2), 300–333. https://doi.org/10.1016/j.cognition.2006.09.011

Vigliocco, G., Vinson, D. P., Druks, J., Barber, H., & Cappa, S. F. (2011). Nouns and verbs in the brain: A review of behavioural, electrophysiological,

neuropsychological and imaging studies. *Neuroscience & Biobehavioral Reviews*, *35*(3), 407–426. https://doi.org/10.1016/j.neubiorev.2010.04.007

Yadav, H., Husain, S., & Futrell, R. (2019). Are formal restrictions on crossing dependencies epiphenominal? *Proceedings of the 18th International Workshop on Treebanks and Linguistic Theories*, 2–12.
